# Sequence-specific dynamics of DNA response elements and their flanking sites regulate the recognition by AP-1 transcription factors

**DOI:** 10.1093/nar/gkab691

**Published:** 2021-08-13

**Authors:** Johanna Hörberg, Kevin Moreau, Markus J Tamás, Anna Reymer

**Affiliations:** Department of Chemistry and Molecular Biology, University of Gothenburg, Gothenburg 40530, Sweden; Department of Chemistry and Molecular Biology, University of Gothenburg, Gothenburg 40530, Sweden; Department of Chemistry and Molecular Biology, University of Gothenburg, Gothenburg 40530, Sweden; Department of Chemistry and Molecular Biology, University of Gothenburg, Gothenburg 40530, Sweden

## Abstract

Activator proteins 1 (AP-1) comprise one of the largest families of eukaryotic basic leucine zipper transcription factors. Despite advances in the characterization of AP-1 DNA-binding sites, our ability to predict new binding sites and explain how the proteins achieve different gene expression levels remains limited. Here we address the role of sequence-specific DNA flexibility for stability and specific binding of AP-1 factors, using microsecond-long molecular dynamics simulations. As a model system, we employ yeast AP-1 factor Yap1 binding to three different response elements from two genetic environments. Our data show that Yap1 actively exploits the sequence-specific flexibility of DNA within the response element to form stable protein–DNA complexes. The stability also depends on the four to six flanking nucleotides, adjacent to the response elements. The flanking sequences modulate the conformational adaptability of the response element, making it more shape-efficient to form specific contacts with the protein. Bioinformatics analysis of differential expression of the studied genes supports our conclusions: the stability of Yap1–DNA complexes, modulated by the flanking environment, influences the gene expression levels. Our results provide new insights into mechanisms of protein–DNA recognition and the biological regulation of gene expression levels in eukaryotes.

## Graphical Abstract

**Graphical Abstract:**
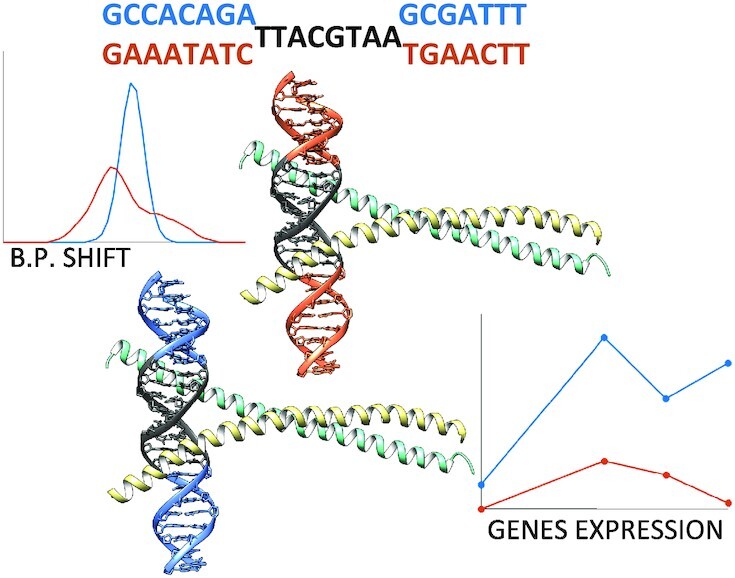
Activator proteins 1 (AP-1) comprise one of the largest families of eukaryotic basic leucine zipper transcription factors (TFs). We show that Ap1 TFs exploit the sequence-specific flexibility of DNA within the response element to form stable protein–DNA complexes. The complex stability also depends on the four to six nucleotides, flanking AP-1 response elements. Bioinformatics analysis of differential expression of the studied genes supports our conclusions: the stability of protein–DNA complexes, modulated by the flanking environment, influences the gene expression levels.

## INTRODUCTION

Activator proteins 1 (AP-1) comprise one of the largest and most evolutionary conserved families of transcription factor proteins (TFs) in eukaryotes, which regulate among other cellular stress responses, cell differentiation and cell proliferation (1,2). The AP-1 factors constitute a subgroup of basic leucine zippers (BZIPs) (3), which control gene transcription by binding as homo- or heterodimers (2,4,5) to specific DNA targets known as AP-1 response elements (ARE). The AP-1 proteins achieve their DNA selectivity predominantly through a direct readout mechanism, when a highly conserved five-residues-motif of the protein basic region, **N**XX**AA**XX**CR**, recognizes the ARE-DNA half-site (3). Despite the high sequence homology of the basic regions, AP-1 factors recognize diverse pseudo-palindromic and palindromic AREs, as well as emergent sites containing only a consensus-like half-site (3,5–7). The lengths of AP-1 response elements also vary. Commonly, AP-1 proteins recognize DNA targets of seven or eight base pairs (bp), where the experimentally derived consensus sequence corresponds to a 7 bp pseudo-palindrome, TGACTCA (3). However, several members of the AP-1 family show preference for longer AREs of 13–14 bp (6,8,9). This diversity of ARE-sequences hampers our understanding of how AP-1 factors achieve their DNA binding selectivity, and consequently the transcription regulation specificity.

The binding of AP-1 factors induce no major DNA deformation (3,10), as observed in experimentally solved structures of AP-1 protein–DNA complexes, however the proteins can potentially exploit local sequence-specific structural variations of DNA (11). The early analysis of crystallographic structures of protein–DNA complexes, by Olsson and colleagues (12), proposed that the most polymorphic pyrimidine(Y)-purine(R) dinucleotide steps could act as flexible ‘hinges’ facilitating the conformational adjustment of DNA to its protein-partner. However, due to the limited number of protein–DNA structures, the study focused on the nearest-neighbour effects. Progress in atomistic molecular dynamics (MD) and in the force fields for nucleic acids, provided further insights into the role of DNA sequence-specific flexibility for protein recognition (13). Microsecond-long MD simulations of DNA oligomers by ABC consortium (14,15), confirmed that certain dinucleotides (YR and RR) oscillate between conformational substates. The studies reported: the local DNA polymorphism is highly heterogeneous and sequence-specific, and is coupled to the tetranucleotide (14,16) or even hexanucleotide level (15), depending both on the nucleotide composition of the central dinucleotide step and its flanking environment. As a result, once bound by a protein the local plasticity of DNA can finalize the DNA transition into a bioactive conformation, as reported by an extensive MD study by Orozco and colleagues (17). Including DNA shape parameters have also shown to improve the accuracy of sequence based predictive models of TF binding selectivity (18).

Another important, but much less explored aspect that can modulate the specific binding of AP-1 factors, and other transcription factor families (19,20), is flanking sequences outside the response elements. The high-throughput study by Steger and colleagues (21) focusing on another subfamily of BZIPs, CREBP transcription factors, showed a strong preference for 5′-R and 3′-Y flanking nucleotides, directly adjacent to the response elements. Bansal and colleagues (22) further highlighted the importance of the flanking regions for several transcription factors, including the BZIP proteins Fos-Jun (which prefer 5′-RR and 3′-YY-flanks) and NFIL3 (which prefer YR for both 5′- and 3′-flanks). Both studies proposed that DNA shape readout could contribute to BZIP-proteins binding specificity, since only nonspecific contacts with the DNA backbone of the flanking nucleotides were found in the crystal structures of the BZIP-DNA complexes. These studies provide evidence that the flanking sequences could play a regulatory role in transcription factors specific binding to DNA, but the underlying molecular mechanism and the number of contributing flanking nucleotides remains to be discovered.

In this paper, we explore mechanistic aspects of the AP-1-DNA recognition process, focusing on the local DNA sequence-specific flexibility, variations in the response elements and flanking environments, using atomistic molecular dynamics simulation in the microsecond range. With DNA flexibility we refer to local sequence-specific variations in DNA helical parameters. As a model system, we employ Yap1 transcription factor (7), a member of *Saccharomyces cerevisiae* AP-1 family, which regulates genes involved in oxidative stress responses and cell detoxification. Differently from AP-1 transcription factors in other eukaryotic organisms, the basic region of Yap1 contains glutamine and phenylalanine instead of alanine and cysteine (**N**xxAQxx**F**R) (3). The phenylalanine presence suggests an increased preference for 5′-TT dinucleotide step at the extremities of Yap1 response element (3,7). However, the response elements identified in promoters of Yap1-modulated genes remain diverse: (7,23–27) TTACTAA, TTACGTAA, TGA(C/G)TAA, T(T/G)ACAAA, etc. For our computational study, we select three different Yap1 response elements from promoter regions of two genes, to account for a variation in the flanking sequences. We observe that the local variations of the helical parameters, shift and slide, within the DNA response element modulate the binding specificity of Yap1. Our data also show that four to six nucleotides, flanking the response elements, influence how easily shift and, to a lesser extent, slide can adjust within the response element to achieve the bioactive conformation upon the protein binding. This sequence-specific flexibility, regulated by the flanking environment, we hypothesize, play a unique role in the recognition process as well as in the stability of the protein–DNA complexes, modulating the firing strength of the corresponding promoters. Our mechanistic findings correlate well with the expression levels of the two selected genes, and of the model-reporter genes containing one instance of the selected Yap1 response elements.

## MATERIALS AND METHODS

### Systems studied

We study 12 systems: Yap1 bound to three different response elements (YRE1: TTACTAA, YRE2: TTACGTAA, YRE3: TGACAAA) in two genomic environments (ATR1, OYE2) (28), as well as unbound DNA in B-form for each corresponding system. The native DNA sequences are obtained from the NCBI database (29) (see Supplementary Figure S1 for their position relative to the transcription starting site) with the Gene IDs 854924 and 856584 for ATR1 and OYE2, respectively. The six studied 23-mer DNA sequences are listed in Table 1.

**Table 1.** The six studied 23-mer DNA sequences in two genomic environments (ATR1 and OYE2) containing Yap1-response elements in bold

Name	Sequences
YRE1_ATR1	TATAGTGA**TTACTAAT**GGAATGG
YRE1_OYE2	GTTTTGCT**TTACTAA**GCACACGA
YRE2_ATR1	GCCACAGA**TTACGTAA**GCGATTT
YRE2_OYE2	GAAATATC**TTACGTAA**TGAACTT
YRE3_ATR1	TGATTATA**TGACAAA**GTTGAGGG
YRE3_OYE2	GCTAGCGA**TGACAAA**ATGTCTCC

### Homology modelling

The Yap1 homodimer is derived through homology modelling in YASARA (30). The Fasta file for the Yap1 BZIP domain (residues 63–130), obtained from Uniprot (31) with ID P19880, is subjected to the comparative modelling tool in YASARA, using the high resolution crystal structure of Pap1 (PDB ID: 1GD2) (3), the closest orthologue of Yap1, as a template. Sequence alignment is shown in Supplementary Figure S2. Quality assessment of the Yap1 homology model, performed by YASARA, where the derived model obtained an optimal score, indicates a high-resolution structure. For the complete parameter sets used for the homology modelling see Supplementary Table S1.

### Protein–DNA docking

To derive the six Yap1–DNA complexes, protein–DNA docking is performed using HDOCK webserver (32). For the docking, Yap1 is defined as the receptor and B-DNA, containing the response elements (TTACTAA, TTACGTAA, TGACAAA) surrounded by two adjacent 5′- and 3′-flanking nucleotides (NNYRENN) of the native environments (ATR1, OYE2), is defined as the ligand. Using default settings positioned Yap1 to enable interactions with the response elements among the top-10 scored complexes. For each Yap1–DNA docking run, we select the highest scored complex among the top-10 docking decoys which positions YRE symmetrically to the two Yap1 monomers (Supplementary Figure S4A–C). We then use the modelling program JUMNA (33) to extend the flanking sites to derive 23-oligomers and remove major Yap1–DNA clashes. The Yap1–DNA docked complexes and the JUMNA processed complexes, used as the starting structures for MD simulations, are provided as separated SI files. For Yap1–YRE2–ATR1 and Yap1–YRE2–OYE2 systems, we also construct complexes using as the ligand the DNA structure derived from the Pap1-DNA complex structure (PDB ID: 1GD2) (3). See additional information on the verification of the docking procedure in supplementary information (Supplementary Figures S3 and S4).

### Molecular dynamics simulations

All molecular dynamics (MD) simulations are performed using the MD engine GROMACS v2018.1 (34). For each simulation a combination of AMBER 14SB (35) and Parmbc1 (36) force fields is used for the protein and DNA, respectively. For Yap1–YRE2–ATR1 system, additional simulation is performed with Cufix force field corrections for DNA (37). The Yap1–DNA complexes and free DNA oligomers are separately solvated in triclinic rectangular periodic boxes by SPC/E water molecules (38) and TIP3P water (38) for the Cufix simulation with a buffer distance of 15 Å to the walls. Each system is neutralized by K + counterions. Additional K + and Cl- ions are then added to reach a physiological salt-concentration of 150 mM. Applying periodic boundary conditions, each system is subjected to energy minimization with 5000 steps of steepest descent, followed by 500 ps equilibration-runs with week position restraints on heavy solute atoms (1000 kJ/mol) in the NVT and NPT ensembles, adjusting temperature and pressure to 300 K and 1 atm. Releasing the restraints, 1.1 μs simulations are then carried out at constant pressure and temperature (1 atm and 300 K).

### Trajectory analysis

The generated trajectories are processed using CPPTRAJ program (39) from AMBERTOOLS 16 software package. The first 100 ns of each trajectory are discarded as equilibration. Subsequently, Curves+, Canal, and Canion programs (40) are used to derive the helical parameters, backbone torsional angles, groove geometry parameters, and ion distributions for each trajectory snapshot extracted every ps. CPPTRAJ is used to derive Yap1–DNA contacts as previously described (9,11). Free energy analysis for each system is performed using the MMPBSA/MMGBSA plugin in AMBERTOOLS 16.

### BZIP-DNA helical parameters motifs

Crystal structures of BZIP-DNA complexes (Supplementary Table S4) bound to different response elements (TGACTCA, TGACGTCA, TTACGTAA) are downloaded from the Protein Data Bank (41), and analysed for helical parameters for the BZIP-DNA complexes and their corresponding naked DNA in B-form by Curves+ (40).

### Differential expression data mining

The differential expression levels of the two Yap1 target genes, ATR1 and OYE2, across different time-points are derived from the study by Salin *et al.* (42). The data are downloaded from the ArrayExpress website through the access number E-TABM-439. The expression level profiles for the two genes are derived by calculating the log2 ratio between WT and the *yap1Δ* mutant under the selenite stress conditions.

### YRE analysis on random promoters’ study

Data for randomly generated promoters with associated expression levels are derived from the study by Boer *et al.* (43), obtained from NCBI’s gene expression omnibus under the access number GSE104878. Internally developed R-scripts are used to isolate promoters possessing only one instance of YRE decorated with at least two (**NN-**YRE-**NN**) or four (**NNNN-**YRE-**NNNN**) of the adjacent 5′- and 3′-flanking nucleotides from the ATR1 or OYE2-environments, and to analyse the associated expression data.

### Additional information

MatLab software is used for the post-processing and plotting of all data. USCF Chimera (44) is used for creating the molecular graphics.

Further details of the methods used are provided in the supplementary information.

## RESULTS AND DISCUSSION

### Yap1–DNA contacts

To address the molecular mechanism of DNA recognition by AP-1 transcription factors, we study Yap1 transcription factor binding to three different Yap1 response elements (YRE), YRE1: TTACTAA, YRE2: TTACGTAA, and YRE3: TGACAAA. To identify the role of the flanking environment outside the response elements, we extract DNA sequences from two Yap1-regulated genes, ATR1 and OYE2, which contain all three YREs (Supplementary Figure S1). To design the 3D structure of the DNA-binding BZIP-domain of Yap1, we use the homology modelling approach with the high-resolution crystallographic structure of Pap1 (PDB ID: 1GD2) (3) the closest orthologue of Yap1 as the template. Yap1 and Pap1 share 39.7% of sequence identity and 56% of sequence similarity within the BZIP domain, with only a two residues difference in the DNA-binding region and identical positions of Leu-residues that secure the dimerization of the factors (Supplementary Figure S2), which justifies the choice of the template and ensures the quality of the model. To derive the structures of Yap1–DNA complexes, we perform macromolecular docking with HDOCK webserver (32), which uses a hybrid algorithm of template-based modelling and *ab initio* free docking. To generate the dynamic profiles of macromolecular interactions and assess the complexes stability, we subject each of the Yap1–YRE systems and the corresponding naked B-DNA oligonucleotides to a microsecond-long all-atom MD simulation, using AMBER 14SB (35) and Parmbc1 (36) force fields for the protein and DNA, respectively. To exclude any bias of the selected force field on the DNA-protein interactions, in particular, the electrostatic description of the negatively charged DNA backbone, we perform for the YRE2–ATR1 system an additional simulation using Cufix force field corrections (37).

To validate that a microsecond-long MD simulation is sufficient for the complexed DNA to reach its bioactive conformation starting from B-DNA, we perform an additional set of simulations for Yap1–YRE2–ATR1 and Yap1–YRE2–OYE2 systems, where the YRE2–DNA structure is adapted from the crystal structure of the complex with Pap1(PDB ID: 1GD2) (3). We then collect average structures over increasing time windows starting from 400ns (Supplementary Table S2, Figure S5), and compare the DNA structures from the simulations starting from B-DNA and from deformed DNA. The RMSD values for Yap1–YRE2–ATR1 (max 1.8 Å) and Yap1–YRE2–OYE2 (max 2.5Å) suggest that a microsecond-long MD simulation is sufficient for docked B-DNA, as the starting state, to converge towards the deformed bioactive conformation.

To assure the reproducibility of our study we also run an additional replica for each of Yap1–ATR1 systems. Our simulations show good convergence according to RMSD (Supplementary Figure S6A and B) and the total number of protein–DNA contacts (Supplementary Figure S7A and B). In all simulations, the RMSD plots show that Yap1 reduces the fluctuations of DNA making it more rigid. In terms of the two ATR1 replicas, we observe similar fluctuations in RMSD, the total number and the nature of protein–DNA contacts for the three different YREs. In addition, the two replicas converge to the same average structure (Supplementary Figure S8).

**Figure 1.**
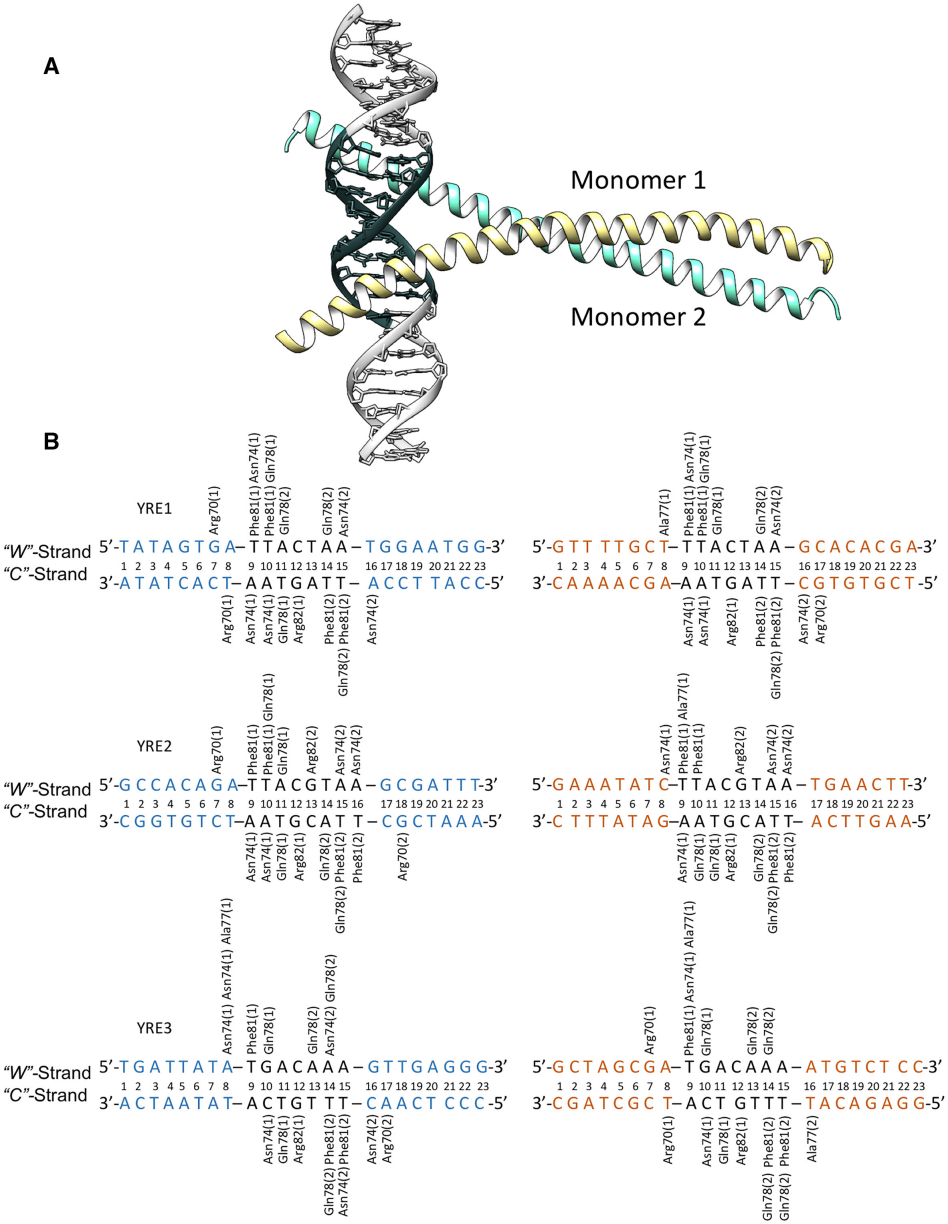
(**A**) Homology model of Yap1 (monomer1 in yellow, monomer 2 in turquoise) bound to DNA, where YRE is highlighted with dark grey. (**B**) Specific contacts between Yap1 and the three YREs (YRE1: TTACTAA, YRE2: TTACGTAA, YRE3: TGACAAA) in two genomic environments (ATR1: blue, OYE2: orange) observed in one microsecond long MD simulations.

Our simulations show that, in analogy to other AP-1 transcription factors, Yap1 utilizes the five-residues-motif of the basic region (**N**xx**AQ**xx**FR**):(3) Asn74, Ala77, Gln78, Phe81 and Arg82 to form the bases-specific contacts with the three DNA response elements (Figure 1). We observe both similarities and differences in the specific contacts formed by Yap1, depending on the YRE and its flanking sites. The similarities include the contacts by Phe81, which recognizes the outer 5′-TT/TG step; by Arg82, which favours the central (C)G bp/bp step; and by Ala77, which interacts with the first thymine of the outer 5′-TT/TG step. The differences include the contacts formed by Asn74 and Gln78 that exhibit a considerable variation in the specific contacts within the three YREs, which are also monomer specific. For instance, Gln78 forms a number of hydrogen bonds with the TA/GA step (T**TA**CTAA, T**TA**CGTAA, and T**GA**CAAA) and its complementary TA/CT step on the opposite strand. Asn74 forms either monodentate/bidentate contacts with the outer 3′-AA step (TTACT**AA**, TTACGT**AA** and TGACA**AA**), or cross-bridging contacts involving cytosine of the 3′-CA step and thymine of the 5′-TG step on the opposite strand (**T**GACAAA/TTTGT**C**A). The scope of the hydrogen bond contacts exploited by Asn74 and Gln78 suggests a compelling molecular mechanism explaining how Yap1 can recognize a variety of YREs. Furthermore, for the systems that are flanked by a 5′-GA/GC/AC step (Watson strand: YRE1–ATR1, YRE2–ATR1 and YRE3–OYE2. Crick strand: YRE1–OYE2, YRE2–ATR1 and YRE3–ATR1) we observe an additional specific contact by Arg70 (**R**xxxNxxAQxxFR), a semi-conserved residue among the BZIP-families. For further details of the intermolecular contacts, see Figure 1, for the time-evolution of the intermolecular contacts, see the dynamic contacts maps (Supplementary Figures S9–S11).

To further analyse the contacts-dynamics of protein–DNA complexation, we perform the clustering of the conformational substates for the residues involved in the specific contacts. The derived cluster maps (Supplementary Figure S12) mirror the fluctuations in the dynamic contacts maps (Supplementary Figures S9-S11). In particular, for Arg70 (**R**xxxNxxAQxxFR) for the systems where the flanks specifically interact with the residue, in YRE1–ATR1/OYE2, YRE2–ATR1, YRE3–ATR1/OYE2 systems, we observe fluctuations between the contacts with the YRE-flanking bases and the DNA backbone (Supplementary Figure S16). For Arg82 (NxxAQxxF**R**), in YRE1–ATR1 and YRE1–OYE2 systems, clustering shows the appearance of the specific Arg82-G contact with the central CG bp (TTA**C**TAA) towards the end of the trajectories (Supplementary Figure S15). For YRE1–ATR1 Phe81 of monomer 2 — the increase of the Phe81-T specific contact (**T**TACGTAA) towards the second half of the trajectory. For YRE2–OYE2 Gln78 of monomer 1 — fluctuations of its specific contacts with the inner (TT**A**CGTAA) and outer (**T**TACGTAA) region of YRE2. For YRE3–ATR1 Asn74 (**N**xxAQxxFR) of monomer 1 — the loss and gain of Asn74-YRE3 specific contact. These observations agree with the earlier reported trends that long side-chain amino acid residues exhibit conformational substates, alternating between interactions with DNA bases and backbone (45–47), and in this fashion act as protein–DNA recognition switches potentially guiding the association-dissociation processes.

### Impact of DNA flanking environment

**Figure 2.**
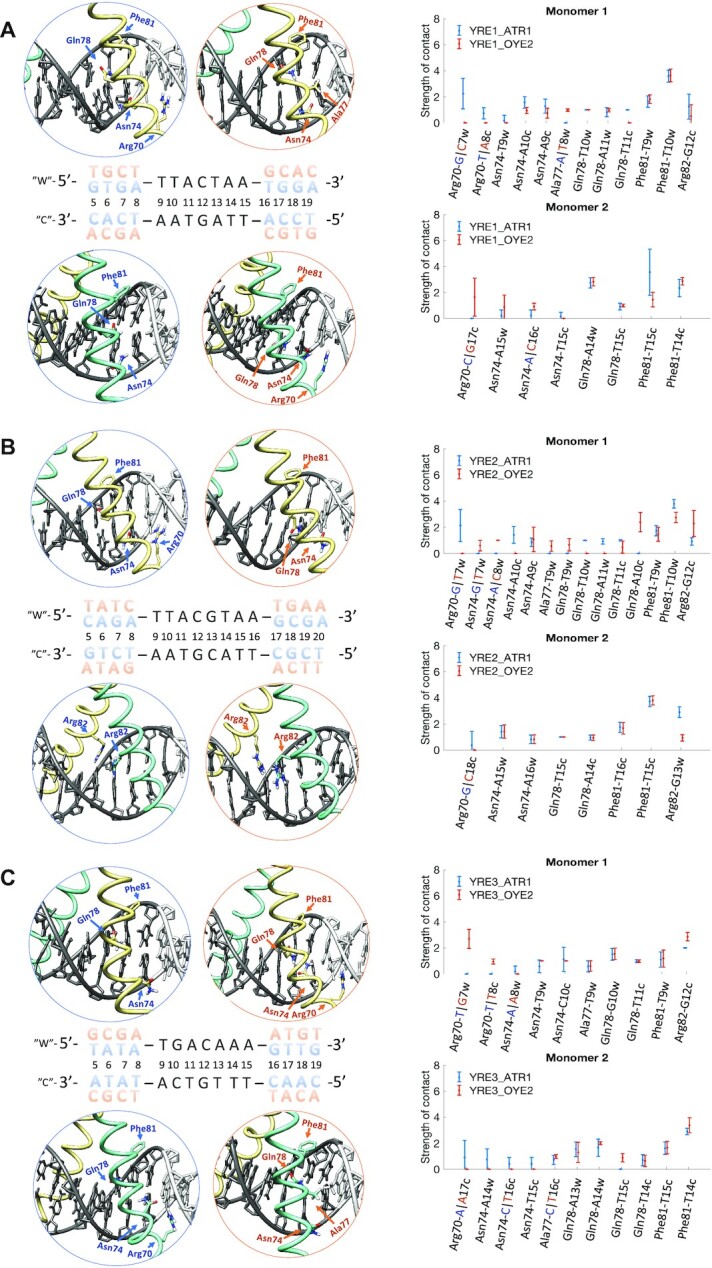
Specific contacts between Yap1 five-residues recognition motif (**R**xxx**N**xx**AQ**xx**FR**) and the three YREs in two genomic environments, ATR1 and OYE2. For the Yap1–DNA complexes, Yap1 monomer 1 is in yellow, Yap1 monomer 2 is in turquoise, and DNA is in grey, where YRE is highlighted with dark-grey. The ATR1-environment is blue-marked and the OYE2-environment is orange-marked. The plots show the strength of specific contacts exploited by Yap1. For the definition of a contact strength see Supplementary Methods. (**A**) Yap1–YRE1. (**B**) Yap1–YRE2. (**C**) Yap1–YRE3.

Our simulations show that both the composition of the response element and the flanking environment impact the specificity of Yap1–DNA contacts (Figure 2A-C and Supplementary Figures S9-S11). Since the Yap1–YRE recognition is dominated by the direct readout mechanism, we hypothesize that the number of specific protein–DNA contacts, namely the contacts between the protein side chains and DNA bases, represents the molecular selectivity. Whereas the number of the total protein–DNA contacts represents the complex stability. To quantitively compare the response elements and assess the role of the flanking environment, we introduce a notion of protein–DNA contacts strength. Namely, we characterize the protein–DNA interactions by pairs of residues, where for each pair we sum up all hydrogen bonds, salt bridges, and hydrophobic (apolar) contacts. For simplicity, the contribution of a single bond of each type is set to 1, since the energy cost varies depending on the nature of interacting atoms, the bond geometry, and the surrounding environment. For time-evolution of the strength of Yap1–DNA contacts, see dynamic contacts maps (Supplementary Figures S9–S11).

**Figure 3.**
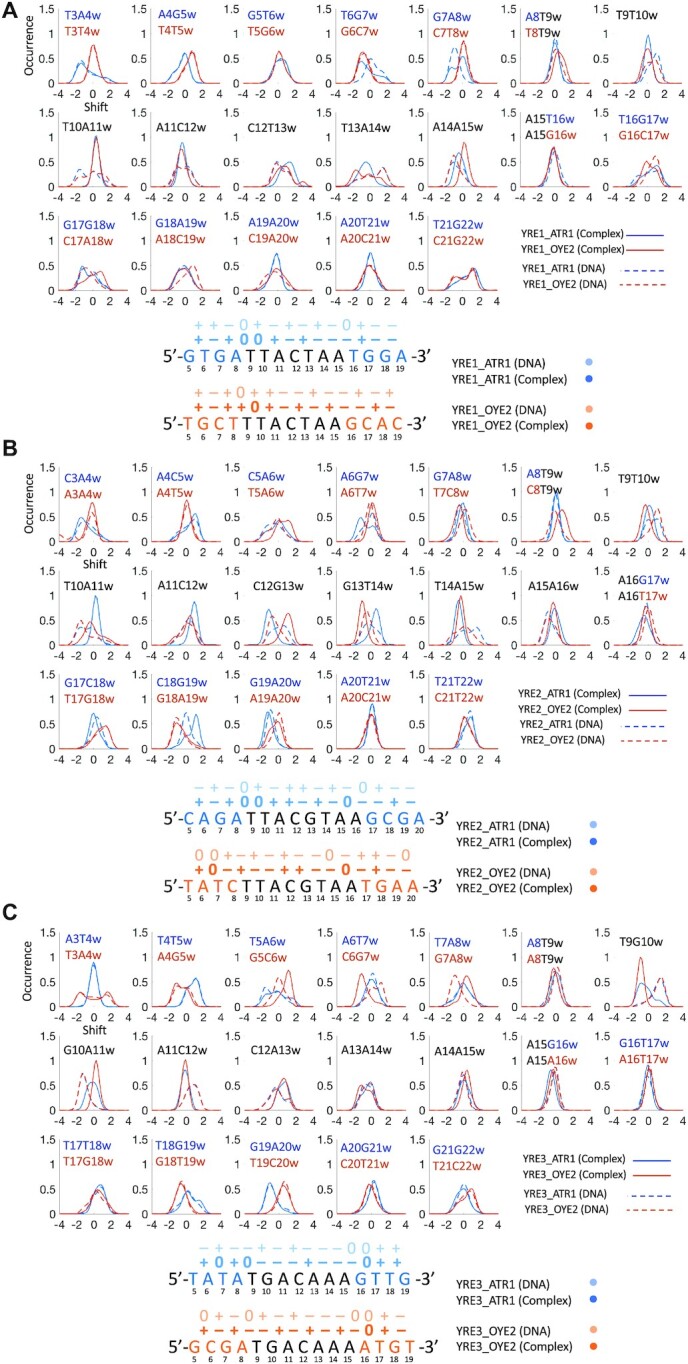
Upper panels: normalised shift distributions for unbound (dashed lines) and Yap1-bound (thick lines) DNA. Bottom panels: most populated shift (the signs ‘–’, ‘+’ and ‘0’ indicate negative, positive and neutral shift) for unbound (light colour) and Yap1-bound (dark colour) for (**A**) YRE1, (**B**) YRE2, (**C**) YRE3. The genomic environments, ATR1 and OYE2, are coloured blue and orange, respectively.

Our simulations show that DNA sequence-specific flexibility modulates the Yap1–DNA recognition process by adjusting the helical and groove parameters to resemble the bioactive conformation (17), facilitating the transcription factor binding. For the major groove binders like Yap1, and BZIP factors in general, shift that involves the relative displacement of bp steps in and out of the major groove, and slide that involves the relative displacement of bp between the backbones of two DNA strands, are the key parameters that enable the formation of specific protein side chains–DNA bases contacts. Our data show that Yap1 forms an overall similar number of contacts with the three YREs, but the flanking environment impacts the strengths of the protein–DNA contacts. When analysing the helical parameters for both Yap1-bound and unbound DNA, we observe changes in shift, slide, and twist in the presence of Yap1 (see the corresponding distributions, presented in the 5′→ 3′ direction, in Figure 3A-C, Supplementary Figure S13A-C, S14A-C, S15A-B, S16A-D). Our data show alterations in helical parameters of up to the nearest four (shift, slide) to six (twist) flanking nucleotides upon Yap1-binding. This in turn modulates the helical parameters distributions within the response elements for Yap1-bound DNA, fine-tuning the direct read-out mechanism. In particular, we observe that broad shift and slide distributions within the response elements result in either the loss or rearrangements of specific contacts. We, therefore, propose a molecular mechanism where DNA sequence-specific flexibility of the flanking sites facilitates the induced fit of the response element for the transcription factor to form stable protein–DNA contacts.

In particular, when comparing Yap1 interactions with pseudo-palindromic YRE1 (TTACTAA) (Figure 2A, Supplementary Figure S9), we observe that the ATR1-environment contributes with an additional Arg70-G specific contact with the 5′-GA flanking step (**GA**-TTACTAA). The rearrangement of shift of the four nearest 5′-flanking bp steps (**GTGA**-TTACTAA) in Yap1-bound DNA (direction 5′→ 3′, Figure 3A, Supplementary Figure S16B), which shifts the GC bp out of the major groove (positive shift, Figure 3A), provides an optimal environment for the contact. Arg70-G specific contact acts as an anchor for monomer 1, placing the monomer deeper within the DNA major groove, which stabilizes the specific contacts with the YRE1 half-site by the five-residues-motif (**N**xx**AQ**xx**FR**), resulting in stronger Asn74-YRE1 and Arg82-YRE1 specific contacts. In contrast, in the OYE2-environment, the four nearest 5′-flanking bp steps (**TGCT**-TTACTAA) are rigid and exhibit similar shift distributions for bound and unbound DNA, which prevents the deep association of Yap1 monomer 1, explaining weaker Asn74-YRE1 and Arg82-YRE1 specific contacts. The positive shift of the CT step (TTA**CT**AA) favours Arg82-G specific interactions (Crick strand: TTA**G**TAA). Though the CT step shows a broad shift distribution, the positive shift population represents the presence of Arg82-G contact (∼50% for ATR1 and ∼30% for OYE2, Supplementary Figure S9A and S15A-B). Both genomic environments result in similar narrow shift distributions for the TAC region of the first YRE1 half-site (T**TAC**TAA), resulting in similarly exploited specific contacts by Gln78 and Phe81 of monomer 1.

For Yap1 monomer 2 that interacts with the second YRE1 half-site, we observe more distinct differences in the specific contacts between the two genomic environments (Figure 2A), linked to the variations in the b.p. shift distributions (Figure 3A). The Y..R (C-**TA**-A) environment makes the TA step of the second half-site more flexible, compared to the first half-site in the Y..Y (T-**TA**-C) environment. This contributes to broad shift distributions of the TA and AA steps, which triggers the conformational change of Yap1 monomer 2, where the α-helix slides along the major groove to preserve Phe81 contacts with the TT step on the Crick strand (**TT**AGTAA). This in turn repositions Gln78 towards the outer AA step (TTACT**AA**) and consequently reduces the strength of Asn74 specific contact (Supplementary Figure S9A). This conformational change is less pronounced in the OYE2-environment, where YRE1 is stabilised by the GCAC 3′-flanking steps (TTACTAA-**GCAC**), which alter their shift to favour Asn74-C and Arg70-G specific contacts (Supplementary Figure S16B) on the Crick strand. There are also similarities: both genomic environments result in similar Gln78-YRE1 contacts, however, not symmetric with respect to the Gln78 contacts of monomer 1.

For palindromic YRE2 (TTACGTAA), we observe greater differences in specific contacts exploited by Yap1 monomer 1 than by monomer 2 (Figure 2B, Supplementary Figure S10). In the ATR1-environment, the rearrangement of shift for the four nearest 5′-flanking bp steps (**CAGA**-TTACTAA) for Yap1-bound DNA (Figure 3B, Supplementary Figure S16C) is similar to YRE1–ATR1. This provides an additional Arg70-G specific contact and positions monomer 1 deeper into the major groove of YRE2 half-site, following the pattern of intermolecular contacts described for YRE1–ATR1-Yap1 monomer 1. In contrast, in the OYE2-environment, the four nearest 5′-flanking bp steps (**TATC**-TTACGTAA) limit Yap1 to restrain shift values within YRE2, which changes the binding preferences of Asn74 and Gln78. The OYE2-environment also contributes to weaker Phe81-T interactions compared to YRE2–ATR1 (Figure 2B, Supplementary Figure S10).

In the case of monomer 2, the 3′-flanking sites of both genomic environments contribute to similar shift distributions for the second YRE2 half-site, resulting in the identical contacts network for DNA-Yap1 monomer 2 (Figure 2B), involving residues Asn74, Gln78 and Phe81. Moreover, YRE2 is a palindrome with a central CG step, which allows Arg82-G specific contacts by both monomers (TTA**CG**TAA). Our simulations indicate, however, that only one monomer exhibits strong specific Arg82-G interactions, while the other monomer forms mainly salt bridge contacts between Arg82 and the DNA backbone (Figure 2B). The shift of the central CG and GT steps (TTA**CGT**AA), which depends on the flanking environment, determines which Yap1 monomer exhibits Arg82-G specific contact: the ATR1-environment promotes strong Arg82(2)-G_‘w’_ interactions and weaker Arg82(1)-G_‘c’_ interactions (indices ‘w’ and ‘c’ indicate Watson and Crick DNA strands, respectively), the reverse trend is observed for the OYE2-environment.

In the additional Cufix (37) simulation, performed for the YRE2–ATR1 system, we notice that Yap1 monomer 1 becomes slightly more dynamic (Supplementary Figure S17C), as a result of stronger fluctuations of Arg70 specific contact (Supplementary Figure S17A), which analogously to the standard simulation (when using AMBER 14SB (35) and Parmbc1 (36) force fields) assures a deeper placement of Yap1 within the DNA major groove. This simultaneously results in stronger fluctuations of the contacts formed by Asn74, Phe81, and Arg82. However, we believe these results are linked to the dynamic behaviour and the flickering power of Arg70 rather than to Cufix force field corrections.

For YRE3 first half-site (**TGA**CAAA) that interacts with Yap1 monomer 1, we observe that the two genomic environments allow similar specific contacts formed by Ala77, Gln78, and Phe81 (Figure 2C and Supplementary Figure S11). The rearrangement of shift (Figure 3C) of the four nearest 5′-flanking bp steps (**GCGA**-TGACAAA) in the OYE2-environment, similar to YRE1–ATR1 and YRE2-ATR1, stabilizes Arg70-G specific contact, and subsequently strengthens Asn74-YRE3 and Arg82-YRE3 specific contacts. In contrast, in the ATR1-environment, the 5′-flanking site is more rigid (**TATA**-TGACAAA), which restricts shift adjustments for the first YRE3 half-site (**TGA**CAAA) in the presence of Yap1. The broad shift distributions induce sliding and bending of Yap1 monomer 1 basic region to maintain the Phe81-T interactions, which in turn increases fluctuations of the Asn74-YRE3 contacts (Figure 2C and Supplementary Figure S11A). YRE3-DNA, which contains TG instead of the TT step (**TG**ACAAA), shows a clear reduction in the specific contacts strength exhibited by Phe81 of monomer 1. However, the reduced shift of the TG step (**TG**ACAAA) allows increasing the strength of the Phe81-T nonspecific contacts (Supplementary Figure S11B). Additionally, in both genomic environments, the shift values of the central AC and CA steps enable Arg82-G specific contact from the Crick strand, stronger compared to YRE1 (Figure 2A, C, Supplementary Figures S9A and S11).

For monomer 2 that interacts with the second half of YRE3 (TGA**CAAA**), we observe similar Gln78-YRE3 and Phe81-YRE3 contacts in both genomic environments (Figure 2C and Supplementary Figure S11). Overall, the second half-site shows no major alterations of shift distributions for unbound versus Yap1-bound DNA (Figure 3C). However, the thymines on the Crick strand (**TTT**GTCA) result in either large fluctuations (ATR1) or loss (OYE2) of specific contacts exploited by Asn74 (Figure 2C). In the OYE2-environment, the presence of a stiff 3′-ATGT flanking sequence contributes to a negative shift of the extended AA step (TGACAA**A-A**T), allowing for an additional hydrophobic contact, Ala77-T (A**T**-TTTGTCA) on the Crick strand. This contact acts as a steric barrier, preventing the deeper placement of Yap1 monomer 2 within the major groove, explaining the loss of Asn74-YRE3 interactions. In the ATR1-environment, the intermolecular interactions are stabilized by an adjacent 3′-GTTG flanking sequence, where the shift values of the flanking steps promote Asn74-C and Arg70-A specific contacts with the flanking AC step on the Crick strand (CA**AC**-TTTGTCA) (Figure 2C and Supplementary Figure S11A). However, Arg70 specific contact with the flanking 5′-AC step is weaker compared to 5′-GC-Arg70 or 5′-GA-Arg70 contracts, described above.

Our data allow differentiating between high and low affinity response elements flanking sequences. In accordance with a recent HT-SELLEX and bioinformatics study (22) focusing on the BZIP factors Fos-Jun and NFIL3, we show that the flanking 5′-GA step promotes stable selective DNA-Yap1 binding. The flanking 5′-TA step, which is listed as high affinity for the NFIL3 factor, is less favourable for Yap1; thus, indicating that *at least* two adjacent flanking nucleotides that surrounds the response element can modulate the selectivity among different BZIP factors. We recognise the flanking sites containing a flanking 5′-T nucleotide as low affinity for Yap1, similarly to what was observed for the Fos-Jun and NFIL3 factors. At the same time, we hypothesize that binding of BZIP factors to response elements flanked by 5′-T nucleotides can be beneficial for a cooperative association of multiple transcription factors; the steric hindrance caused by the thymines in the major groove can lower the energy cost for a bending deformation of DNA towards the minor groove facilitating the binding of another transcription factor (48).

Our data indicate that Yap1 utilizes the local sequence-specific flexibility of DNA to form stable protein–DNA contacts. To validate if the proposed mechanism can be characteristic for the BZIP family in general, we analyse DNA helical parameters of available crystal structures of BZIP-DNA complexes covering three different response elements: ‘TGACTCA’, ‘TGACGTCA’, and ‘TTACGTAA’ (Supplementary Figure S23, Table S4). We compare to the reference structure: B-DNA created with the modelling program JUMNA (33), which accounts for sequence-specific polymorphic effects. Although, the statistics is limited to only 12 structures, we observe, consistent with our findings, that helical shift is altered within the response elements upon BZIP factors binding. We notice differences in the level of adjustment in DNA helical parameters for the different X-ray structures, though these variations may be linked to the amino-acid composition of the BZIP domains, which may impact how deep the factors can be positioned within the DNA major groove. Also, BZIP factors DNA-binding domains are flexible and can tolerate a certain degree of fluctuations in shift and slide without it strongly affecting the protein–DNA contacts network. Unfortunately, the available experimental structures of BZIP-DNA complexes provide no insight on the impact of the flanking sites as they contain short DNA sequences, including optimal response element for the specific transcription factor.

We also derive shift parameters for each tetranucleotide of the response elements, based on microsecond-long all atomistic molecular dynamics simulations, from the BIGNASim database (49) (http://mmb.irbbarcelona.org/BIGNASim/) to avoid any biased conclusions caused by the choice of the reference structure. We see that all bp steps exhibit high standard deviations. (Supplementary Table S5), however the mean values are significantly different from those upon BZIP binding, which further supports that BZIP factors require local adjustments in shift, and to lesser extend in slide, and that the mechanisms and data we describe herein could be representative for the entire BZIP family.

Furthermore, the available biochemical data supports that the flanking sites are important for the binding capacity of Yap8 and Yap5 (9,50,51), two homologs of Yap1. Particularly interesting is Yap5, which has shown to activate the CCC1 gene by binding to only one of the two identical response elements ‘TTACTAA’ present in the promoter region, where the functional and non-functional binding sites differ in the flanking sites nucleotide composition, e.g.: ‘AATA**TTACTAA**CATA’ versus ‘TCAA**TTACTAA**TGTA’, respectively.

Additionally, we observe an alteration of twist for Yap1-bound DNA, as changes in shift are coupled to changes in twist through DNA backbone BI → BII transitions (14,52,53). This observation is interesting in the context of DNA supercoiling and its role in eukaryotic transcriptional control (54). Together with writhe, twist modulates DNA supercoiling transitions along the chromatin fibre. Recently, we have shown that MafB (a member of human BZIP family) (11), asymmetrically changes the sequence-specific response of DNA to torsional stress, making DNA effectively more rigid. The molecular mechanism of the observed phenomenon is based upon MafB forming a number of specific contacts with the torsionally flexible YR dinucleotide steps thus restraining their shift. Instead, more torsionally rigid dinucleotides are forced to absorb the imposed torsion, resulting in an increased energy cost of twisting in comparison with unbound DNA. Therefore, the degree to which shift, and to less extend slide, can adjust upon association with transcription factors will locally modulate the torsional rigidity of DNA. We hypothesise that the increased local torsional rigidity of DNA impacts how long transcription factor bound promoters stay open for transcription, hence regulating transcription initiation rates.

### Ion populations

The ion populations in the DNA major- and minor grooves, can affect the protein–DNA contacts, are coupled to shift/twist transitions in a sequence-specific manner. The sequence-specific differences in ion populations arise from two different effects: (i) the sequence-specific flexibility of DNA backbone and (ii) the sequence-specific positions of electronegative groups in DNA bases (55–57). Here, we monitor the K+-ion populations within the DNA grooves for unbound and Yap1-bound DNA using Canion program (Supplementary Figures S18-S21). We also derive electrostatic potential maps for trajectory-averaged structures of Yap1-bound and unbound DNA using the Poisson Boltzmann solver APBS+PDB2PQR (Supplementary Figure S22A–C) (58).

We observe significant differences in K^+^-ion populations along the flanking sites, which persist in the protein-bound state. This is interesting from a perspective of random N-terminal tails interactions with DNA, in particular in the flanking regions. The majority of BZIP factors, including Yap1, have substantial random coil N-terminal tails, rich in positively charged residues (9). Thus, the observed differences in ion populations could potentially fine-tune the DNA-residence time of the protein. In addition, from our simulations, we observe that an adjacent 5′-GA step provides the more stable Arg70-G interactions compared to a 5′-GC step, even though both flanking steps undergo similar alterations in shift for Yap1-bound DNA to set the environment for the Arg70-contacts. Analysing ion distributions (Supplementary Figure S18), we observe for unbound DNA, consistent with the previous findings (56), that a GC step accumulates a higher K^+^ population in the major groove compared to a GA step. The K^+^ population around the GC step is not completely depleted in the presence of Yap1, resulting in a competition with Arg70-G specific contact. We observe similar response for a GT step, thus explaining the large fluctuations of Arg70-A specific contact with the AC step in YRE3-OYE2. The derived electrostatic potential maps also exhibit sequence-specific areas of high electronegativity in DNA grooves, consistently where the Canion analysis revealed K^+^ population peaks.

### Bioinformatics analysis of model genes expression levels

**Figure 4.**
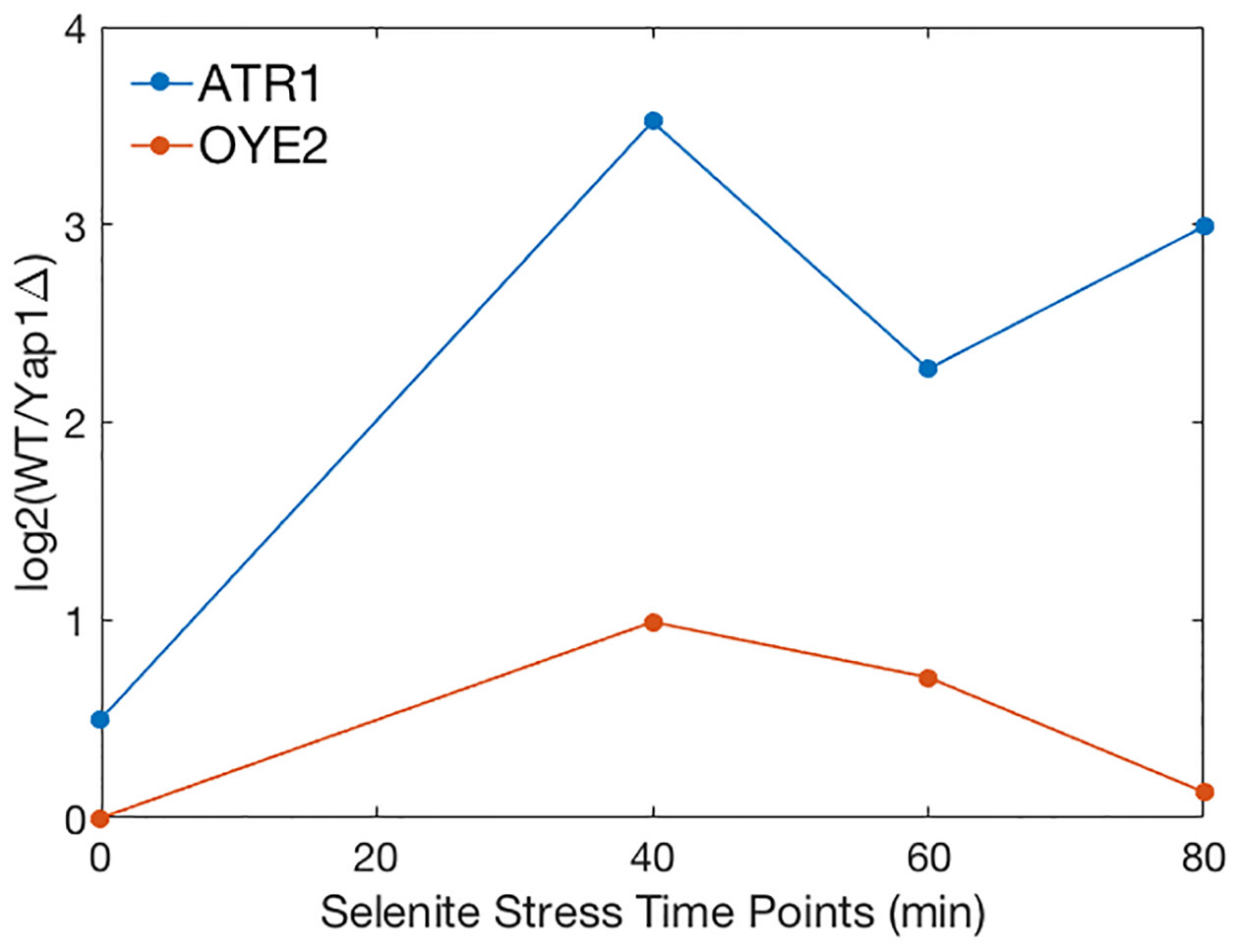
Differential expression levels of Yap1 target genes, ATR1 and OYE2, under selenite stress. Differential expression level between wild type cells (WT) and cells missing YAP1 gene (*yap1Δ*) were obtained from MicroArray quantification at different time points after selenite stress induction.

To relate our hypothesis that the stability of the Yap1–YRE complex modulates the expression levels to experimental data, we analyse the differential expression levels of ATR1 and OYE2 genes of two yeast strains, wild type versus *yap1Δ*, over time under sodium selenite stress that activates Yap1 (42). We see that the ATR1 gene is significantly more differentially expressed compared to OYE2 (Figure 4). In the ATR1-environment, the Yap1 response elements, YRE1 and YRE2, are surrounded by the favouring 5′-[RY/YR]GA flanking sites (RYGA–YRE1 and YRGA–YRE2), which facilitate the desired shift alterations within the repose elements leading to the stable protein–DNA contacts; whereas in the OYE2-environment, only one of the response elements, YRE3, contains this 5′-flanking motif (RYGA–YRE3). In addition, in the OYE2-environment, two of the response elements, YRE1 and YRE3, are surrounded by an adjacent 5′-T nucleotide (for YRE1, Watson strand: **T**TTACTAA; for YRE3, Crick strand: **T**TTTGTCA), which as our simulations show, due to a steric hindrance, resulting in a reduced number of specific contacts. Nevertheless, our simulations imply that adjacent 5′-T flanking nucleotides will not completely abolish Yap1 binding; if the complementary DNA strand contains a more favourable flanking environment. For instance, for YRE3 the OYE2-environment 5′-**GCGA-**TGACAAA, Watson strand, which stabilizes contacts between Yap1 monomer 1 and the first YRE3 half-site. The other YRE3 half-site contains an extended region of thymines (5′-AC**AT-TTT**GTCA, Crick strand), which sterically restricts the deeper association of monomer 2 within the DNA major groove, leading to reduced strength of specific protein–DNA contacts.

To further investigate the role of YREs flanking environment in the regulation of gene expression, we use a library of 100 million randomly generated promoters of 80 bp in length and their associated expression levels, given by a dual reporter gene system (43). The expression levels have been divided into 18 expression bins from the weakest (0) to the strongest (17). We first check the expression levels of the 80 bp-promoters containing only one YRE (and no other known TF-binding motif according to YeTFaSCo database) (59), with the two adjacent 5′-flanking and 3′-flanking nucleotides corresponding to the ATR1- and the OYE2-environments. The corresponding expression levels (Supplementary Figure S24) exhibit a significant variation.

We continue the filtering process and compare the expression levels of the 80 bp-promoters containing four adjacent flanking nucleotides corresponding to the ATR1- and OYE2-environments. For YRE1, the filtering results in one unique instance (ATR1: GTGA**TTACTAA**TGGA; OYE2: TGCT**TTACTAA**GCAC), which shows a stronger expression response in the ATR1-like environment than in the OYE2-like environment, 7 versus 5 in arbitrary expression units. This is consistent with the discussed preference for the flanking 5′-RYGA sequence. Also, for YRE2, the filtering provides one unique instance (ATR1: YRGA**TTACGTAA**GCRR; OYE2: YRTC**TTACGTAA**TGRR), where both promoters show similar associated expression levels around 8 in arbitrary expression units. For YRE3, the filtering found only instances where the flanking sequences resemble but are not identical to the ATR1-environment (YRYR**TGACAAA**RYYR). The associated expression levels of YRE3-like model promoters vary significantly, with an average expression level of 9 and a standard deviation of 5.7. The expression levels for YRE2 and YRE3 do not show a clear correlation to our predictions about the flanking sites and the strength of binding. One possible explanation is that Yap1 contains an 80-residues long N-terminal tail rich in positively charged residues, which was not included in the model, but which could stabilise the protein–DNA interactions. Also, we cannot exclude the possibility that the extracted expression levels also depend on other factors, such as competitive binding by homologous BZIP proteins or cooperative binding by other TFs, etc. In addition, our simulations show that the palindromic nature of YRE2 (TTA**CG**TAA) allows Yap1 to form more specific contacts with DNA, which could explain the lesser sensitivity towards the flanking environments. We also observe for YRE2 that the less favourable 5′-flanking sites in the OYE2-environment result in the rearrangement of Yap1–DNA specific contacts rather than a reduction; this can also contribute to similar expression levels. For YRE3, the high standard deviation in expression levels of the resembling ATR1-environments may suggest an impact of longer than four nucleotides flanking sites. However, based on our simulations we hypothesize that YRE3 can be a cooperative driven/dependent Yap1 response element. The weaker binding of Yap1 to YRE3 compared to YRE1 and YRE2, as discussed, may induce a different structural response in DNA, such as bending towards the minor groove, which could effectively enhance the cooperative binding of transcription factors that can result in an overall stronger transcription response. Hence, the YRE3–ATR1 resembling promoters that show lower expression levels might exhibit weaker cooperative actions between transcription factors.

In attempt to identify the length of the flanking sites for the 80 bp-promoters that impacts the gene expression levels, we group the promoters sequences according to their flanking sites unique nucleotide composition with (i) two nucleotides (NN-YRE[1/2/3]-NN) and (ii) four nucleotides (NNNN-YRE[1/2/3]-NNNN). With the same tight filtering criteria that the promoters sequences should contain only one TF-binding site, the filtering yields 256 unique groups for each YRE for the first set of promoters, with the following total number of promoter sequences: for YRE1 – 17371, YRE2 – 4952, and YRE3 – 11020; and for the second set of promoters, for YRE1 – 14315 groups containing 17371 sequences, YRE2 – 4595 groups and 4952 sequences, and YRE3 – 9644 groups and 11020 sequences, correspondingly. For each unique group containing more than one promoter sequence, we calculate the standard deviations of the expression levels followed by the Student t-test analysis, which shows a significant difference in the distributions of expression level standard deviations between the two selected sets of promoters (Supplementary Figure S25). Hence, the statistical analysis shows that taking four adjacent flanking nucleotides into account compared to two, decreases the average distribution dispersion of the expression levels among the different instances. To include a greater number of flanking nucleotides would produce unique groups containing one, if any, promoter sequence per group, which would make the statistical analysis impossible.

We complement our bioinformatic analysis with MMPBSA/MMGBSA calculations in AMBERTOOLS 16 (Supplementary Table S3). The MMPBSA/MMGBSA approach, although approximate (60), allows to estimate whether enthalpy or entropy drives the Yap1–DNA complexation, and to rank the modelled complexes binding affinities. The calculations show that the enthalpic contributions dominate as expected, since the BZIP-DNA recognition follows the direct read-out mechanism. This is also in accordance with experimental measurements for human AP-1 factors through isothermal titration calorimetry (61). From the enthalpy and entropy, we derive the binding-energies ‘ΔG’ for each Yap1–DNA complex, which, although exhibit high standard deviations (∼18–23 kcal/mol), agree with our conclusions that the ATR1 environment is more favourable.

## CONCLUSION

AP-1 BZIP transcription factors execute their gene regulatory programs through specific binding to the corresponding DNA response elements. The DNA recognition mechanism of AP-1 proteins follows the direct read-out principle, when the highly conserved (Supplementary Figure S26), among the subfamilies, motifs of the proteins basic regions form specific contacts with the bases of the response elements. The response elements recognised by AP-1 vary both in lengths, seven-14 bp, and the nucleotide composition (3,5,6). Moreover, these short specific DNA stretches occur in organisms' genomes much more frequently than the actual genes regulated by the transcription factors, which implies that the complexity of the recognition process goes beyond the ‘simplistic’ direct read-out mechanism. Using microsecond-long all-atom molecular dynamics simulations, we addressed two additional aspects: the roles of DNA sequence-specific flexibility and the response elements' flanking sequences, for the molecular recognition process, protein–DNA complex stability, and in general for the regulation of DNA transcription reaction. As a model system, we employed yeast Yap1 basic leucine zipper transcription factor interacting with three different Yap1 response elements (YRE1: TTACTAA, YRE2: TTACGTAA, YRE3: TGACAAA) from two native genomic environments (ATR1 and OYE2).

Our data show that for the recognition by Yap1, DNA sequence-specific flexibility fine-tunes the direct readout mechanism. Adjustment of DNA base pairs shift and to a lesser extend slide helical parameters within the response element creates an optimal environment in the major groove to allow for stable, specific contacts with the five-residues-motif of Yap1 (**N**xxx**AQ**xx**FR**). Previous MD studies (17) confirm that DNA sequence-specific flexibility facilitates the conformational transition of unbound DNA to its bioactive state, where the transition proceeds more smoothly if the DNA sequence is the corresponding consensus sequence for a particular protein, rather than a random sequence. Our simulations further show that this conformational transition also depends on the flanking environment surrounding the response element, which is not involved in contacts with the protein. Combining our observations with the analysis of the available crystallographic structures of BZIP-DNA complexes (Supplementary Table S4 and Figure S23) suggests that the proposed mechanism of the protein–DNA recognition can be universal for BZIP factors. We observe alterations in helical parameters for four to six flanking nucleotides, which impact how efficiently Yap1 can restrain shift, and to a lesser extend slide, distributions of the bp steps within the response element. Unfavourable flanking sites result in broad shift distributions within YREs, which either causes a reduction or a rearrangement of specific contacts exploited by Yap1. This, we suggest, will influence the binding affinity and allow the transcription factor to discriminate between different genomic locations containing the same response element. Bioinformatics analysis of the available high throughput expression data supports our conclusions. In addition, we know from the previous studies (14,52,53) that change in shift brings changes in twist, important for the regulation of DNA supercoiling transitions and, by extension, transcriptional control. Thus, the level of adjustment of bp shift when a transcription factor is bound to DNA directly translates into DNA local twist flexibility, and consequently the energetic cost of DNA supercoiling transitions (11). The described molecular mechanism, we hypothesise, allows the transcription factor to regulate the opening of gene promoters and subsequently their firing potentials, and by extension, the gene expression levels.

## DATA AVAILABILITY

All data generated and analysed in this study are available from the corresponding author upon request.

## Supplementary Material

gkab691_Supplemental_Files
