# Supplementary material for: Sequence-specific dynamics of DNA response elements and their flanking sites regulate the recognition by AP-1 transcription factors
Source: Nucleic Acids Res. 2021 Aug 13;49(16):9280–93. doi: 10.1093/nar/gkab691 (PMC8450079; doi:10.1093/nar/gkab691)
Supplement: gkab691_Supplemental_Files [file gkab691_supplemental_files.zip › SI_Yap1-DNA_recognition_MD_study_revision3.pdf]

Supporting Information

Sequence-specific dynamics of DNA response elements and their flanking sequences regulates the recognition by AP-1 transcription factors

Johanna Hörberg, Kevin Moreau, Markus J. Tamás, and Anna Reymer*
Department of Chemistry and Molecular Biology, University of Gothenburg, Gothenburg 40530, Sweden
*E-mail: anna.reymer@gu.se.

Methods: Additional Information

Homology Modelling

We subject the sequence (Residue 63-130) of Yap1 BZIP domain to the automatic homology tool in YASARA[1], using Pap1 (PDB 1GD2)[2] as template. Sequence alignment is shown in Figure S2. For the homology modelling, YASARA constructs the Yap1 homology model based on chain F of Pap1 crystal structure. The derived homology model was predicted to be of high quality according to YASARA validation tool.

Benchmark Protein-DNA docking

Benchmarking of HDOCK[3] is performed by first testing the ability of the docking algorithm to recreate the Pap1-DNA crystal structure (PDB ID: 1GD2). For the benchmarking we perform two docking runs where Pap1 is defined as the receptor and B-DNA or the deformed "bioactive" crystal structure DNA is defined as ligand. Using the deformed DNA from the crystal structure as ligand reproduces the Pap1-DNA crystal structure complex among the top-2 best scored decoys (Figure S3A). Using B-DNA as a ligand provides 1-2 decoys among the top-10 scored complexes (Figure S3B) with only slight deviation to the crystal structure (<2Å). This implies that HDOCK can be used to generate high resolution BZIP-DNA complexes.

Benchmark B-DNA as Starting State

Benchmarking of selecting B-DNA as starting state for deriving Yap1-DNA complexes is also performed. For YRE2 (TTACGTAA) we derive Yap1-DNA complexes both (1) through protein-DNA docking using B-DNA as ligand and (2) through superposition to the Pap1-DNA crystal structure to collect the deformed "bioactive" DNA structure. Flanking sites are extended by JUMNA.[4] Microsecond long molecular dynamics simulations show that regardless the starting state of DNA, the Yap1-DNA complexes converge towards the same average structures (Table S2 and Figure S5A-B), indicating that starting from B-DNA is reliable

Molecular Dynamics Simulations: Additional Protocol Details

All molecular dynamics (MD) simulations are performed using the MD engine GROMACS v2018.1.[5] For each simulation a combination of AMBER 14SB[6] and Parmbc1[7] force fields is used for the protein and DNA, respectively. The Yap1-DNA complexes and free DNA oligomers are separately solvated in triclinic rectangular periodic boxes by SPC/E water molecules[8] with a buffer distance of 15 Å to the walls. Each system is neutralized by K+ counterions. Additional K+ and Cl- ions are then added to reach a physiological salt-concentration of 150 mM. Applying periodic boundary conditions, each system is subjected to energy minimization with 5000 steps of steepest descent, followed by 500 ps equilibration-

runs with week position restraints on heavy solute atoms (1000 kJ/mol) in the NVT and NPT ensembles, adjusting temperature and pressure to 300 K and 1 atm. Releasing the restraints, 1.1 microsecond simulations are then carried out at constant pressure and temperature (1 atm and 300 K). Temperature is controlled by a weak-coupling thermostat[9] with a coupling constant of 0.2 ps and pressure is controlled by an isotropic Parrinello-Rahman barostat[10] with a coupling constant of 2 ps. All bonds involving hydrogen atoms are constrained with the LINCS algorithm,[11] allowing the time step of 2 fs. Electrostatic interactions are treated with the Particle Mesh Ewald summation method[12] using a short-range cutoff of 10 Å. The van-der-Waals forces are also truncated at 10 Å with added long-range corrections. The neighbour pair list for nonbonded interactions is updated every 20th step through the Verlet cutoff scheme.[13] Centre of mass movement is removed every 0.2 ps to eliminate translational kinetic energy build-up.[14]

Cufix Corrections

To analyse the dependency of the simulation results on the selected force field, we also run an additional simulation for the YRE2_ATR1 system using Cufix corrections,[15] solvated by TIP3P water,[8] with the same MD set-up as described above.

Contact-Network Analysis

Analysis of the protein-DNA contacts network for the six Yap1-DNA complexes is performed using CPPTRAJ[16] for each trajectory snapshot extracted at 1 ps intervals. Protein-DNA contacts present for less than 10% of the trajectories are excluded. The protein-DNA contacts are characterized by pairs of residues, divided into 'specific', i.e. interactions formed between the protein side chains and DNA bases, and 'non-specific', i.e. interactions formed with at least one of the molecules' backbones. The contacts formed between each protein-DNA residue pair are summed, where for simplicity, the contribution of each contact is set to 1. The distance limit of a hydrogen bond interaction is ≤ 4 Å between the relevant heavy atoms, and the angle limit is ≥ 135° at the intervening hydrogen atom. For a salt bridge, the limit is ≤ 4.0 Å between the end-group nitrogen of lysine and arginine, and the DNA phosphate group. For a hydrophobic contact, the limit is ≤ 6Å between the centre of mass of hydrophobic residues (Ala, Ile, Leu, Met, Phe, Trp, and Cys) and DNA bases. The derived time series of Yap1-DNA interactions allow construction of dynamic contacts maps for specific and non-specific contacts, characterizing the binding specificity and the stability of the Yap1-DNA complexes along the trajectories.

Analysis of Electrostatic Surfaces

Electrostatic surfaces are derived for each YRE-DNA (TTACTAA, TTACGTAA, TGACAAA) in the two genomic environments (ATR1 and OYE2) using PDB2QPR+APBS server (http://server.poissonboltzmann.org).[17] For the PDB2QPR calculations, Force Filed and output naming are set to AMBER. For the APBS "mg-auto options": type of PBE to be solved is set to "nonlinearized"; boundary conditions definition is set to "Multiple Debye-Hückel"; biomolecular dielectric constant is set to 8; biomolecular point charges are mapped by "Quintic B-spline discretization"; number of grid points is changed to 30; and the dielectric ion-accessibility coefficients are modelled by "Molecular Surface Definition".

References

1. Krieger, E. & Vriend, G. YASARA View – molecular graphics for all devices – from smartphones to workstations. *Bioinformatics* **30**, 2981–2982 (2014).
2. Fujii, Y., Shimizu, T., Toda, T., Yanagida, M. & Hakoshima, T. Structural basis for the diversity of DNA recognition by bZIP transcription factors. *Nat. Struct. Biol.* **7**, 889 (2000).
3. Yan, Y., Zhang, D., Zhou, P., Li, B. & Huang, S.-Y. HDOCK: a web server for protein–protein

and protein–DNA/RNA docking based on a hybrid strategy. *Nucleic Acids Res.* **45**, W365–W373 (2017).
4. Lavery, R., Zakrzewska, K. & Sklenar, H. JUMNA (junction minimisation of nucleic acids). *Comput. Phys. Commun.* **91**, 135–158 (1995).
5. Abraham, M. J. *et al.* GROMACS: High performance molecular simulations through multi-level parallelism from laptops to supercomputers. *SoftwareX* **1–2**, 19–25 (2015).
6. Maier, J. A. *et al.* ff14SB: Improving the Accuracy of Protein Side Chain and Backbone Parameters from ff99SB. *J. Chem. Theory Comput.* **11**, 3696–3713 (2015).
7. Ivani, I. *et al.* Parmbsc1: a refined force field for DNA simulations. *Nat. Methods* **13**, 55–58 (2016).
8. Mark, P. & Nilsson, L. Structure and Dynamics of the TIP3P, SPC, and SPC/E Water Models at 298 K. *J. Phys. Chem. A* **105**, 9954–9960 (2001).
9. Berendsen, H. J. C., Postma, J. P. M., van Gunsteren, W. F., DiNola, A. & Haak, J. R. Molecular dynamics with coupling to an external bath. *J. Chem. Phys.* **81**, 3684–3690 (1984).
10. Parrinello, M. & Rahman, A. Polymorphic transitions in single crystals: A new molecular dynamics method. *J. Appl. Phys.* **52**, 7182–7190 (1981).
11. Hess, B., Bekker, H., Berendsen, H. J. C. & Fraaije, J. G. E. M. LINCS: A linear constraint solver for molecular simulations. *J. Comput. Chem.* **18**, 1463–1472 (1997).
12. Darden, T., York, D. & Pedersen, L. Particle mesh Ewald: An N·log(N) method for Ewald sums in large systems. *J. Chem. Phys.* **98**, 10089–10092 (1993).
13. Páll, S. & Hess, B. A flexible algorithm for calculating pair interactions on SIMD architectures. *Comput. Phys. Commun.* **184**, 2641–2650 (2013).
14. Harvey, S. C., Tan, R. K.-Z. & Cheatham III, T. E. The flying ice cube: Velocity rescaling in molecular dynamics leads to violation of energy equipartition. *J. Comput. Chem.* **19**, 726–740 (1998).
15. Yoo, J. On the stability of protein–DNA complexes in molecular dynamics simulations using the CUFIX corrections. *J. Korean Phys. Soc.* (2021) doi:10.1007/s40042-021-00063-9.
16. Roe, D. R. & Cheatham, T. E. PTRAJ and CPPTRAJ: Software for Processing and Analysis of Molecular Dynamics Trajectory Data. *J. Chem. Theory Comput.* **9**, 3084–3095 (2013).
17. Unni, S. *et al.* Web servers and services for electrostatics calculations with APBS and PDB2PQR. *J. Comput. Chem.* **32**, 1488–1491 (2011).
18. de Boer, C. G. *et al.* Deciphering eukaryotic gene-regulatory logic with 100 million random promoters. *Nat. Biotechnol.* **38**, 56–65 (2020).
19. de Boer, C. G. & Hughes, T. R. YeTFaSCo: a database of evaluated yeast transcription factor sequence specificities. *Nucleic Acids Res.* **40**, D169–D179 (2012).

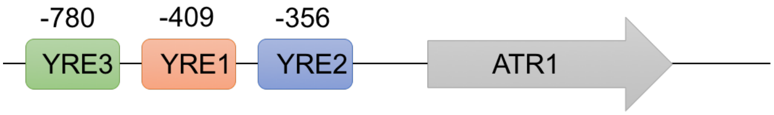

YRE1: TATAGTGA-**TTACTAA**-TGGAATGG
YRE2: GCCACAGA-**TTACGTAA**-GCGATTT
YRE3: TGATTATA-**TGACAAA**-GTTGAGGG

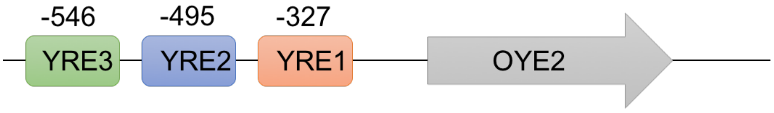

YRE1: GTTTTGCT-**TTACTAA**-GCACACGA
YRE2: GAAATATC-**TTACGTAA**-TGAACTT
YRE3: GCTAGCGA-**TGACAAA**-ATGTCTCC

Figure S1: The three different Yap1 Response Elements (YREs): YRE1 – TTACTAA, YRE2 – TTACGTAA, and YRE3 – TGACAAA, located in the vicinity of the Yap1 regulated genes, ATR1 and OYE2.

```
#=======================================
#
# Aligned_sequences: 2
# 1: Yap1(target)
# 2: Pap1(template)
# Matrix: EBLOSUM62
# Gap_penalty: 10.0
# Extend_penalty: 0.5
#
# Length: 68
# Identity:      27/68 (39.7%)
# Similarity:    38/68 (55.9%)
# Gaps:           6/68 ( 8.8%)
# Score: 117.0
#
#
#=======================================

Yap1(target)       1 DPETKQKRTAQNRAAQRAFRERKERKMKELEKKVQSLESIQQQNEVEATF     50
                         ..||.|||||||||||:|||..:|.||.:|.:|:.:.....:|...
Pap1(template      1 ----SSKRKAQNRAAQRAFRKRKEDHLKALETQVVTLKELHSSTTLENDQ     46

Yap1(target)      51 LRDQLITLVNELKKYRPE     68
                     ||.::..|..||:..:
Pap1(template     47 LRQKVRQLEEELRILK--     62


#---------------------------------------
#---------------------------------------
```

Figure S2: Pairwise sequence alignment for homology modelling of Yap1 (Target) using Pap1 (Template) as template. The Yap1 BZIP domain aligns to the 62 residues of Pap1, with sequence identity and sequence similarity of 39.7% and 55.9%, respectively. The alignment generates six gaps that are positioned outside the DNA binding region, which make them less severe for the purpose of our study. YASARA accounts for the gaps by performing more sophisticated multiple alignments combined with secondary structure prediction.

Table S1: Parameters used for the homology modelling of Yap1 in YASARA.

Homology Modelling Parameters	
Modelling speed (slow=best)	Slow
Number of PSI-BLAST iterations	6
Maximum number of templates to be used	1
Maximum number of templates with same sequence	1
Maximum oligomerization state	4
Maximum number of alignment variations per template	5
Maximum number of conformations tried per loop	50
Maximum number of residues added to the termini	10

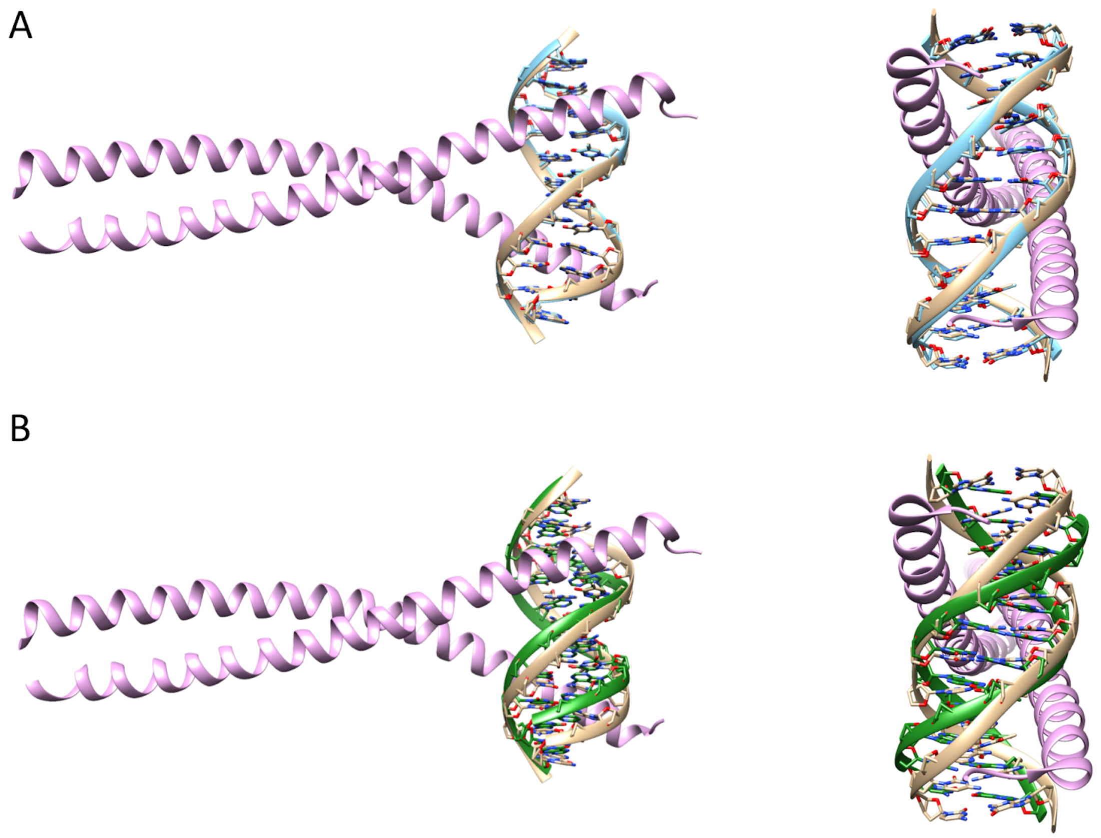

Figure S3: Reproducibility of Pap1-DNA (PDB ID: 1GD2) crystal structure with HDOCK docking server using Pap1 as receptor and **A.** deformed crystal structure DNA (blue) or **B.** B-DNA (green) as ligand.

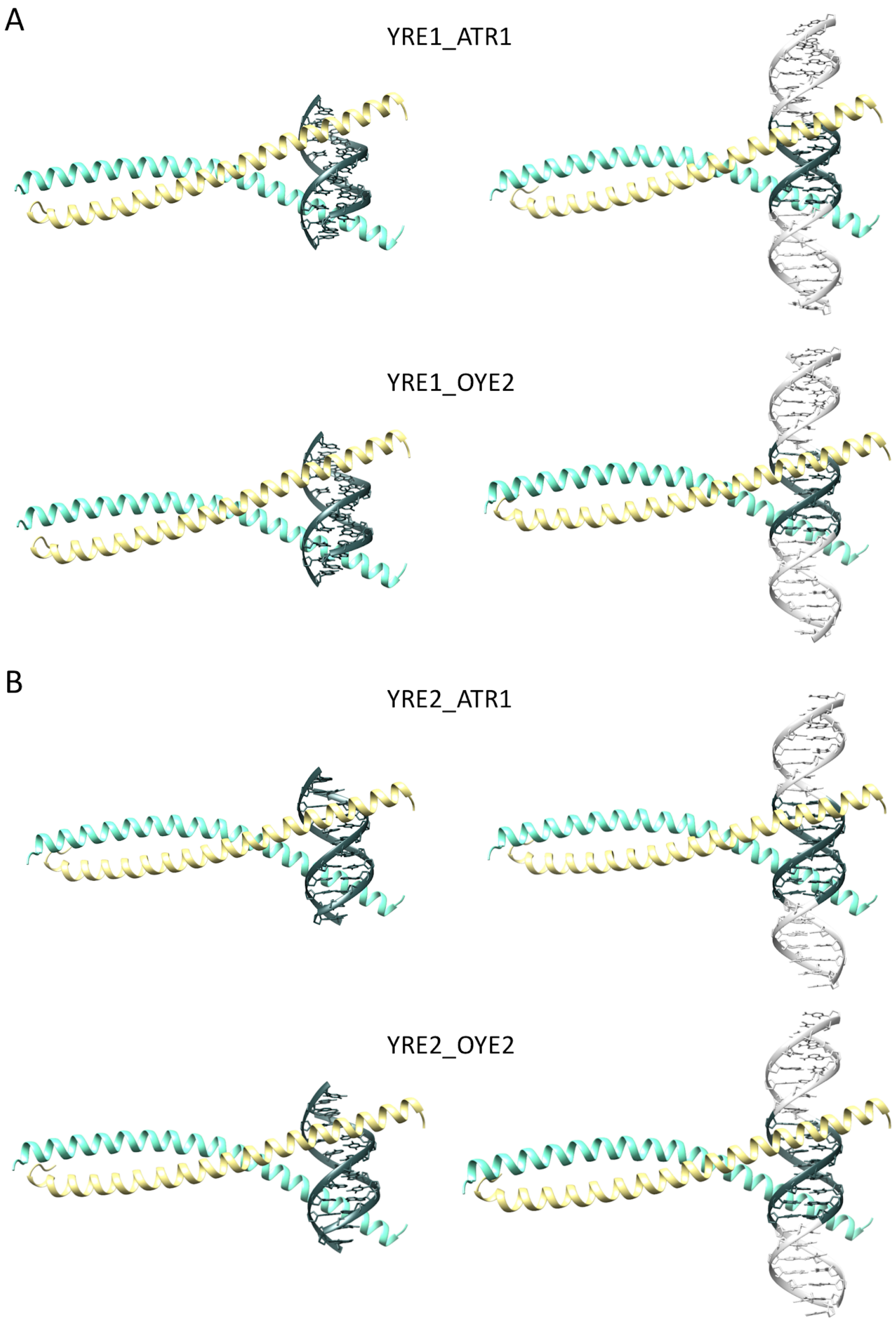
A
YRE1_ATR1
YRE1_OYE2
B
YRE2_ATR1
YRE2_OYE2

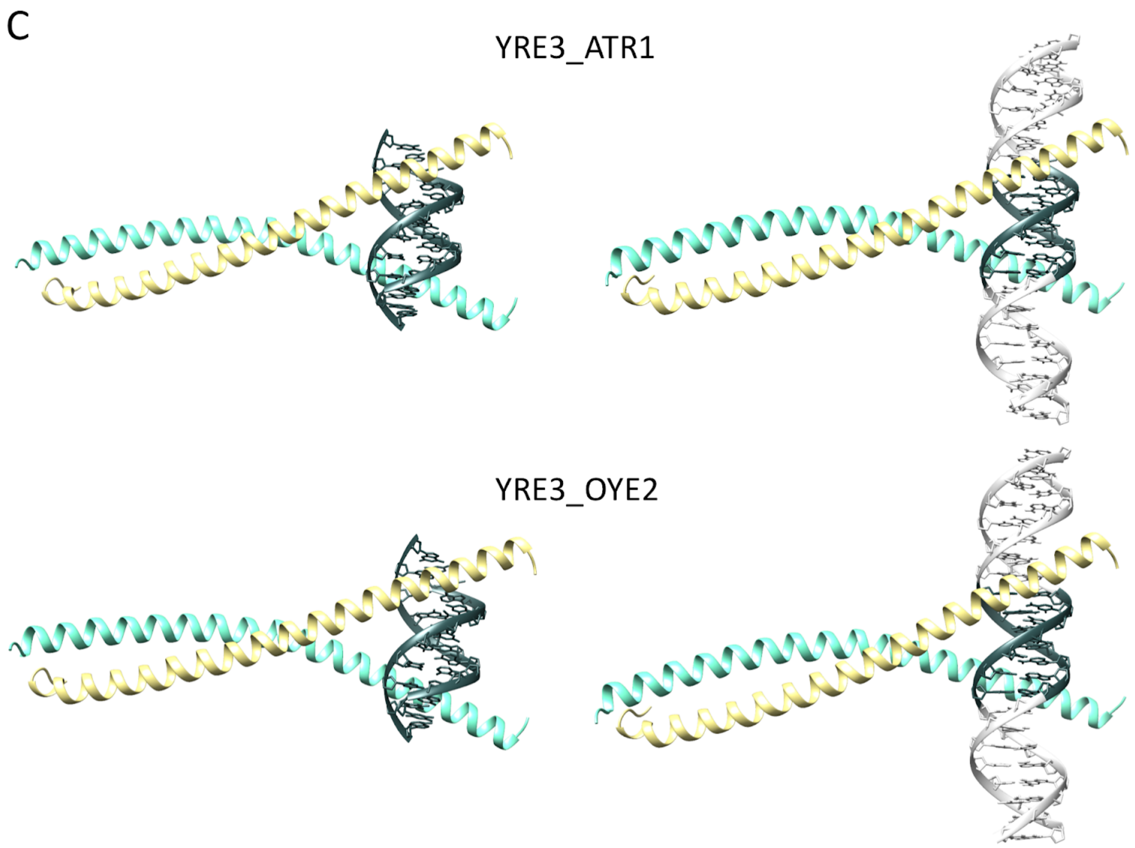

Figure S4: Right-hand panel: derived Yap1-DNA complexes using HDOCK. Left-hand panel: Extension of flanking sites using JUMNA. **A.** YRE1, **B.** YRE2, **C.** YRE3.

Table S2. Differences in RMSD of average structures of Yap1-YRE2 complexes starting from either docked B-DNA state or deformed DNA derived from Pap1 crystal structure.

Average structure time interval	ATR1 RMSD (Å)	OYE2 RMSD (Å)
100-400ns	1.6	2.1
100-500ns	1.8	2.0
100-600ns	1.8	1.7
100-700ns	1.6	2.5
100-800ns	1.5	2.3
100-900ns	1.2	2.1
100-1000ns	1.2	1.4
100-1100ns	1.1	1.4

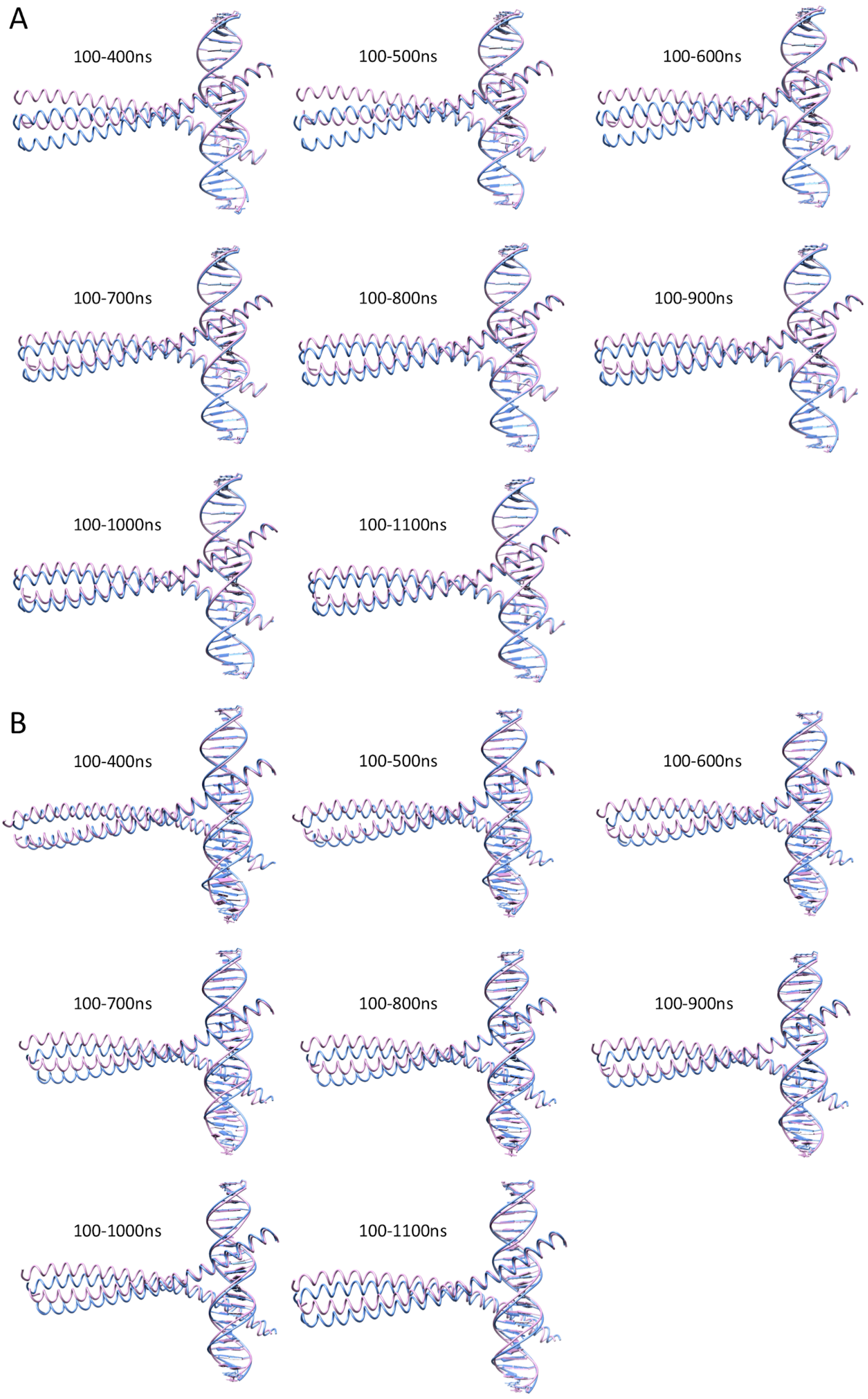

Figure S5: Differences in RMSD of average structures of Yap1-YRE2 complexes starting from either docked B-DNA state (pink) or deformed DNA derived from Pap1 crystal structure (Blue). **A.** ATR1**. B.** OYE2.

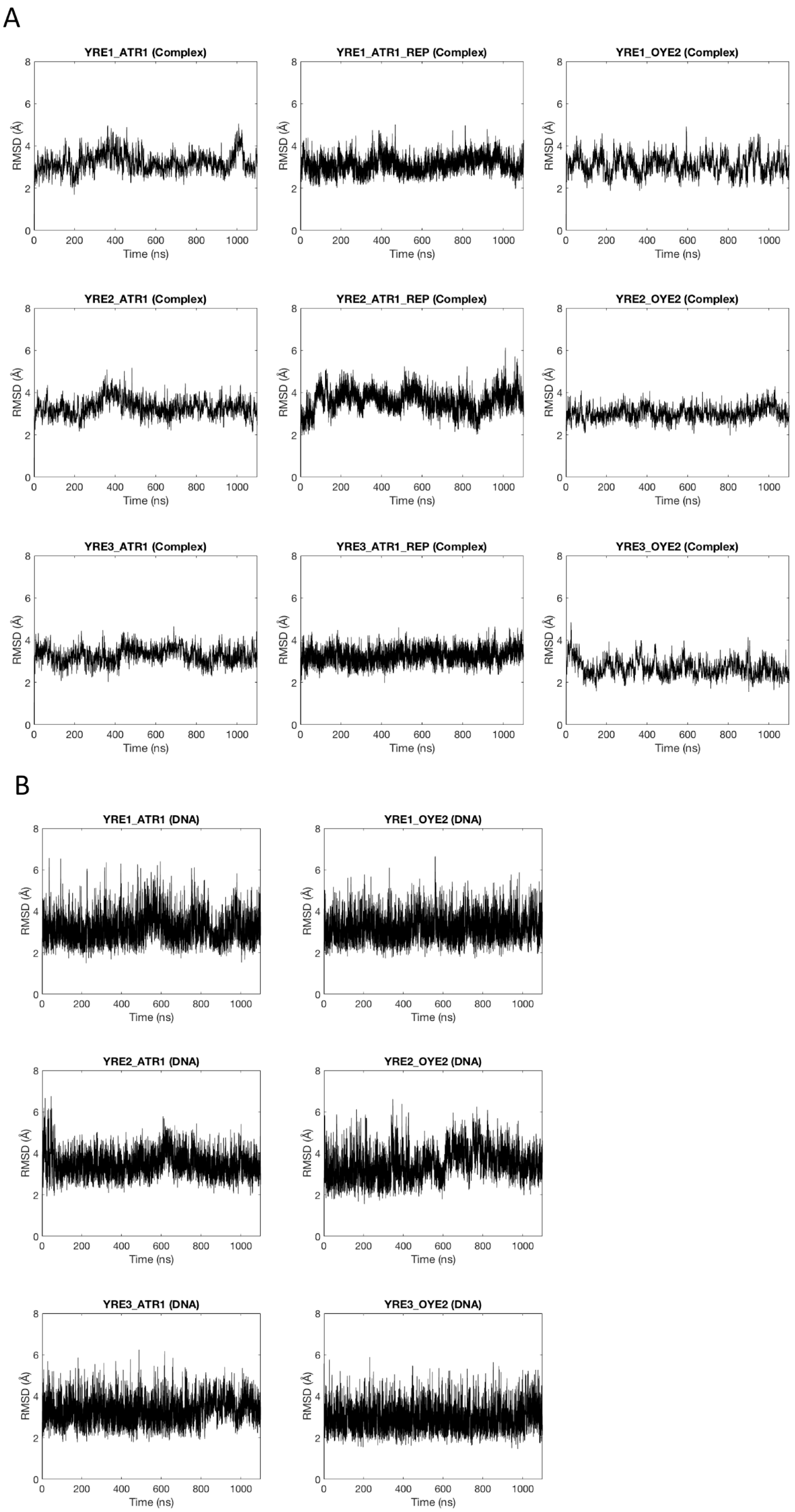

Figure S6: Time-evolution of RMSD for **A.** Yap1-YRE complexes and **B.** Naked DNA.

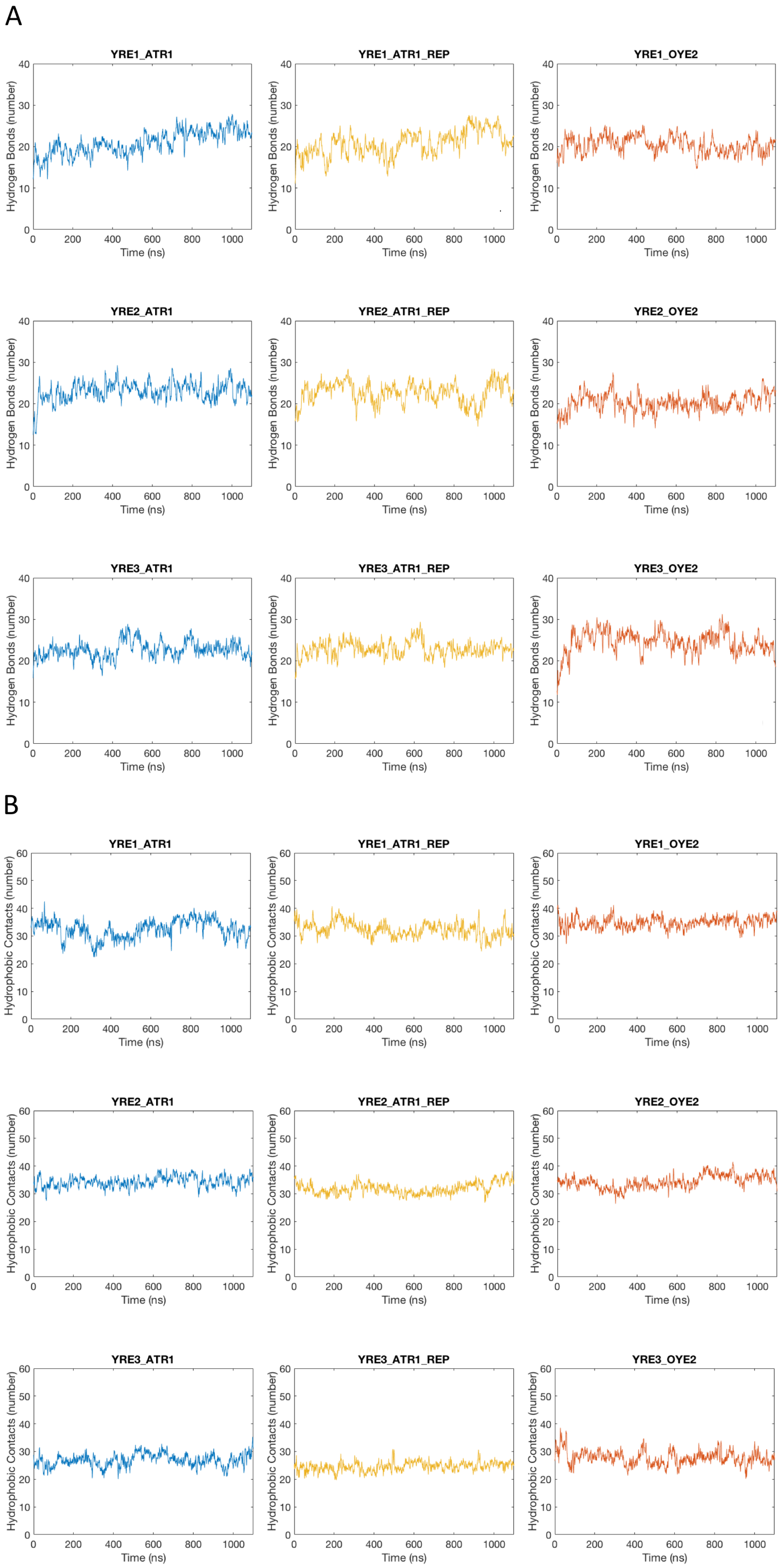

Figure S7: Time-evolution of Yap1-DNA contacts. **A.** Hydrogen bonds and salt-bridges. **B.** Hydrophobic contacts. The two replicas of ATR1 are denoted with blue (Replica 1) and yellow (Replica 2) colours. OYE2 is denoted with orange colour.

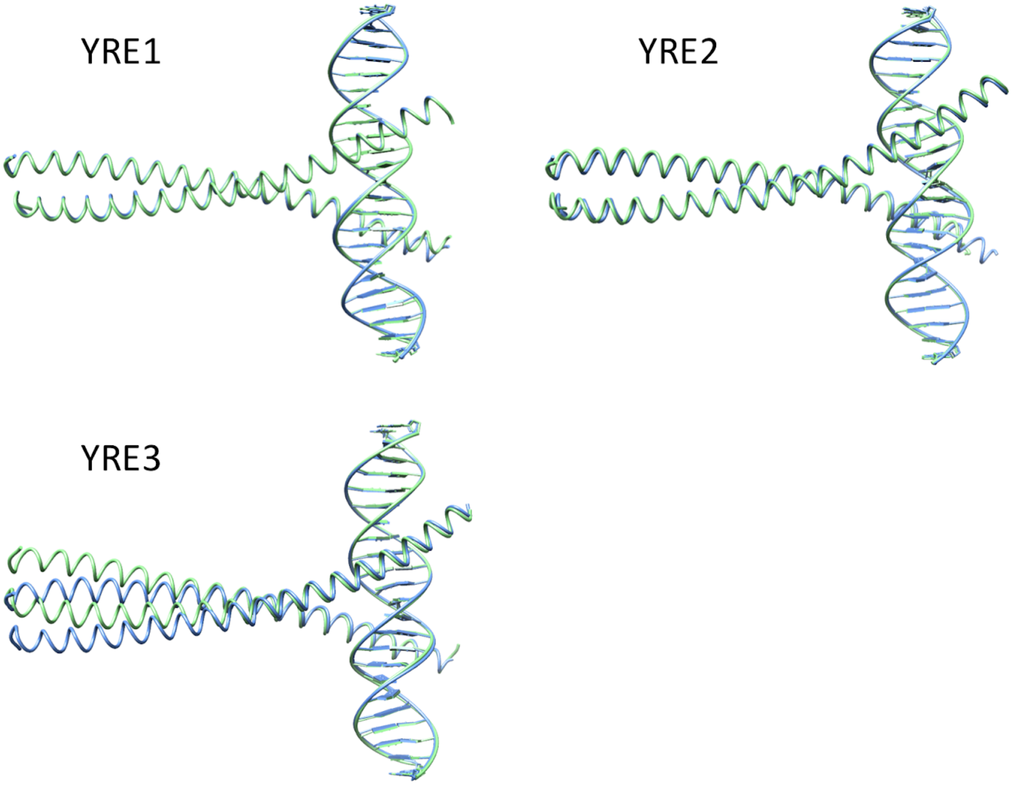

Figure S8: Superposition of 1 microsecond average structures of the two ATR1-replicas; DNA and the DNA binding region of Yap1 show negligible differences.

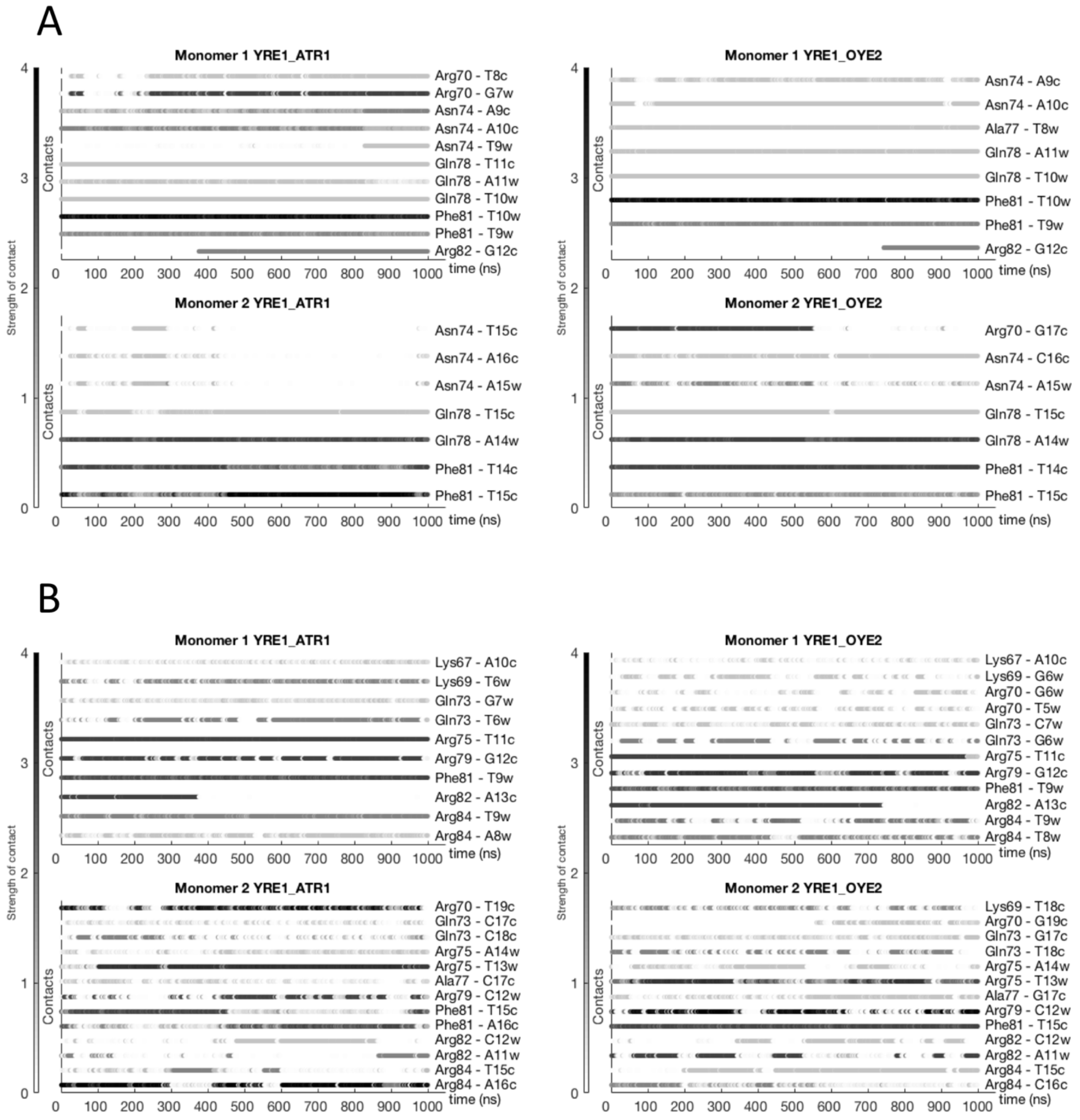

Figure S9: Dynamic interaction maps for Yap1 in complex with YRE1 in the ATR1- and OYE2-environments. The maps illustrate the strength of **A.** specific and **B.** nonspecific Yap1-DNA contacts as a function of time. Indices "w" and "c" refer to Watson- and Crick-DNA strands.

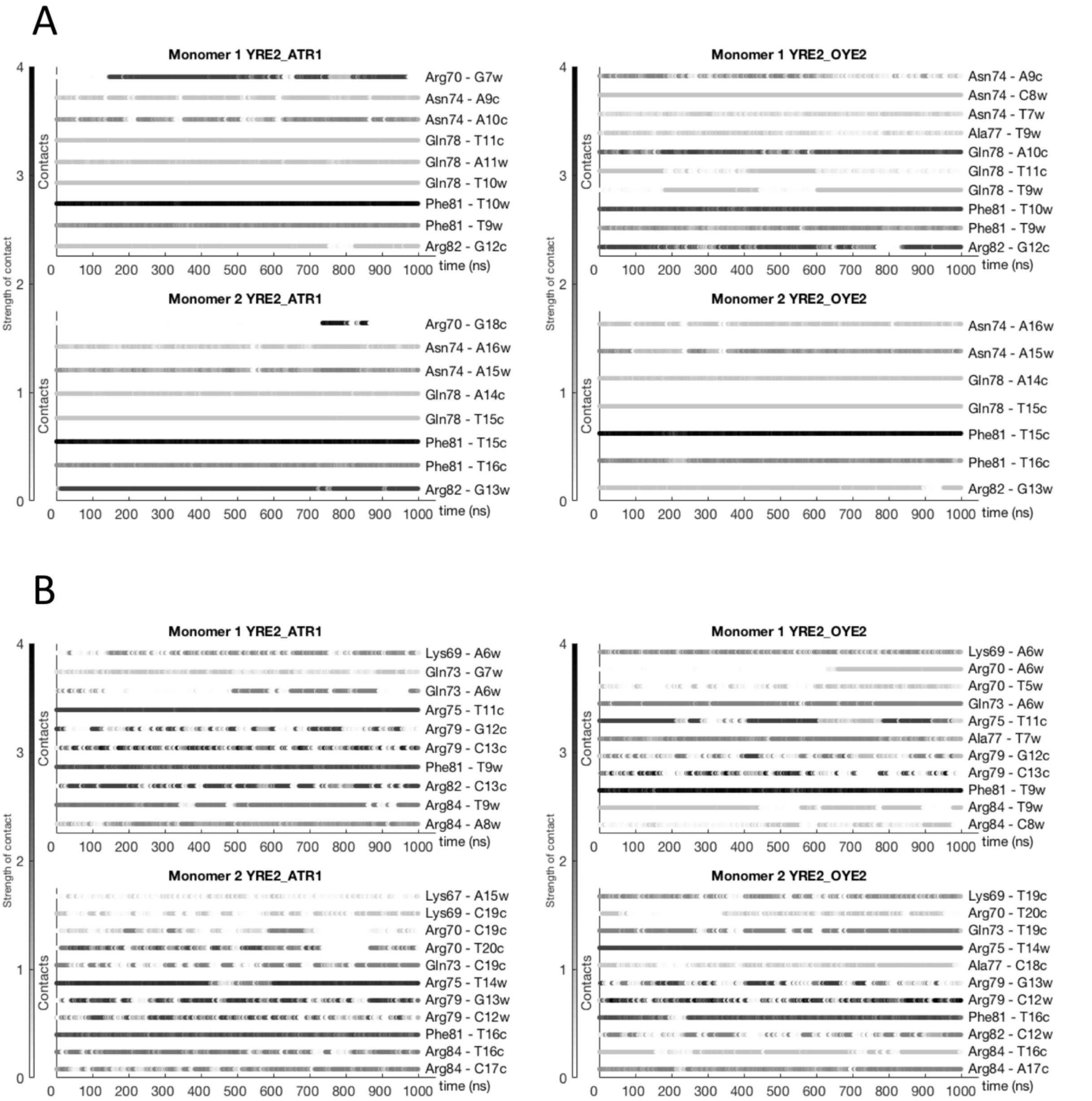

Figure S10: Dynamic interaction maps for Yap1 in complex with YRE2 in the ATR1- and OYE2-environments. The maps illustrate the strength of **A.** specific and **B.** nonspecific Yap1-DNA contacts as a function of time. Indices "w" and "c" refer to Watson- and Crick-DNA strands.

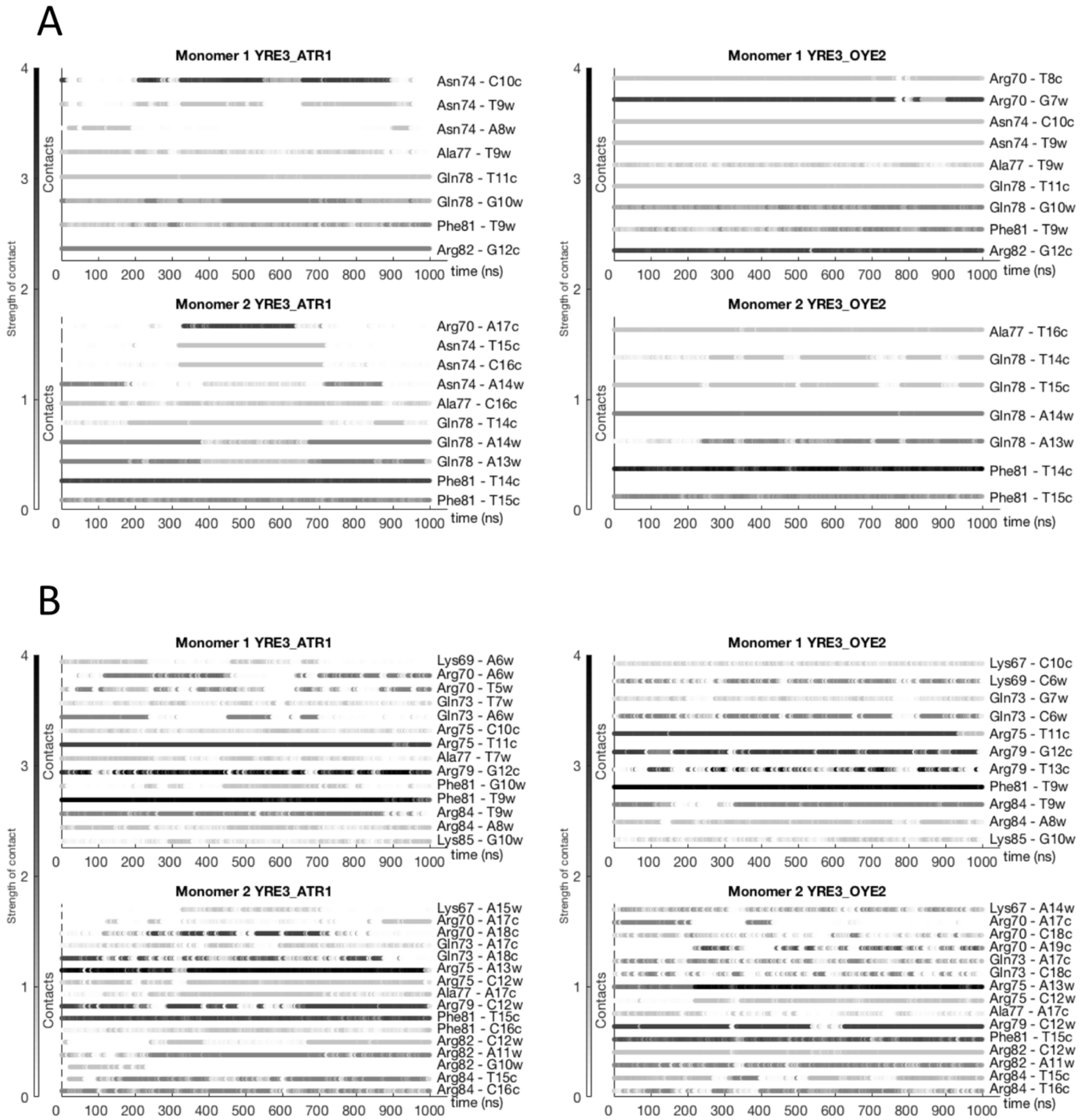

Figure S11: Dynamic interaction maps for Yap1 in complex with YRE3 in the ATR1- and OYE2-environments. The maps illustrate the strength of **A.** specific and **B.** nonspecific Yap1-DNA contacts as a function of time. Indices "w" and "c" refer to Watson- and Crick-DNA strands.

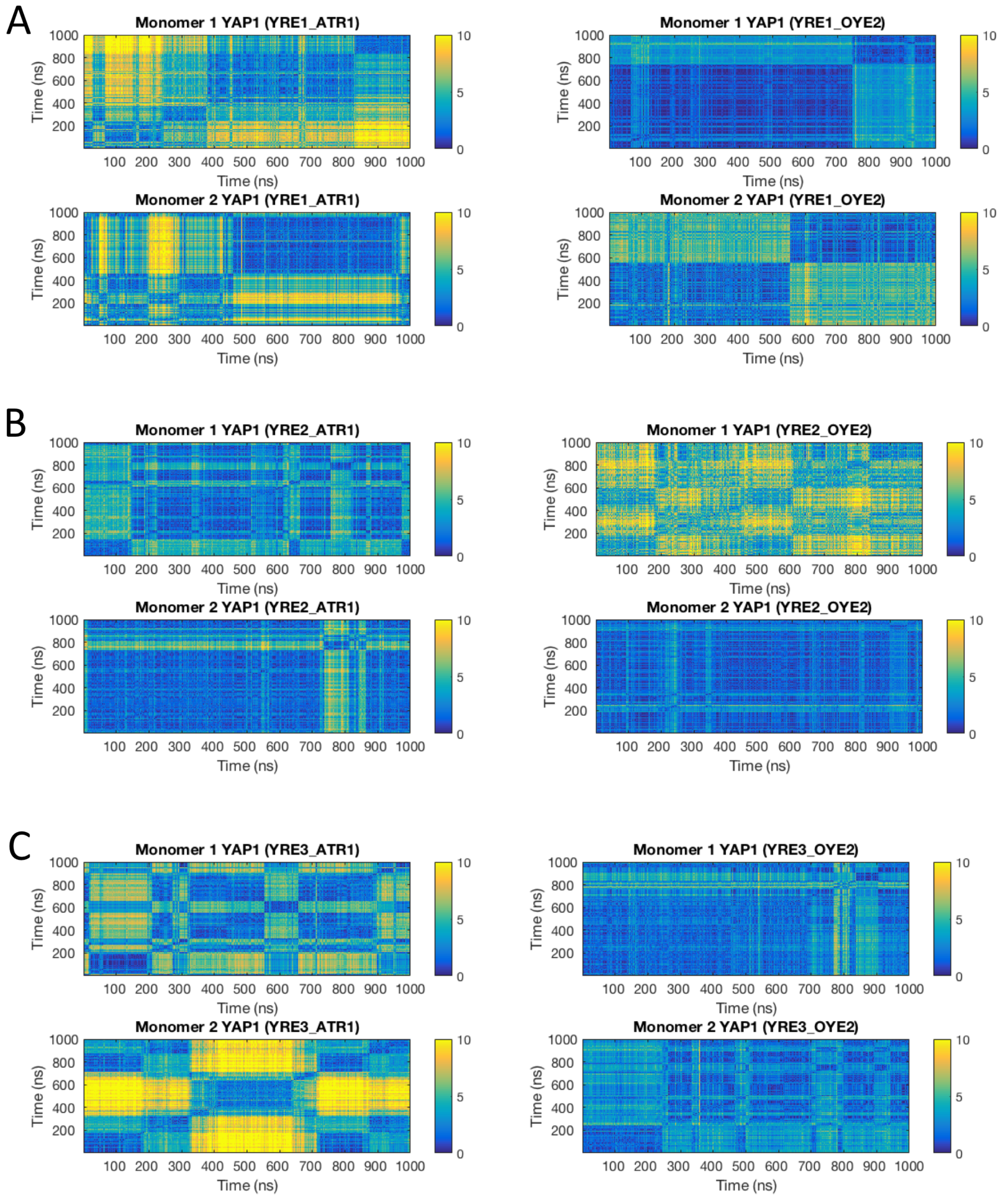

Figure S12: Clustering of Yap1-DNA specific contacts for **A.** YRE1, **B.** YRE2, **C.** YRE3. Colourbar corresponds to pairwise distance between two frames.

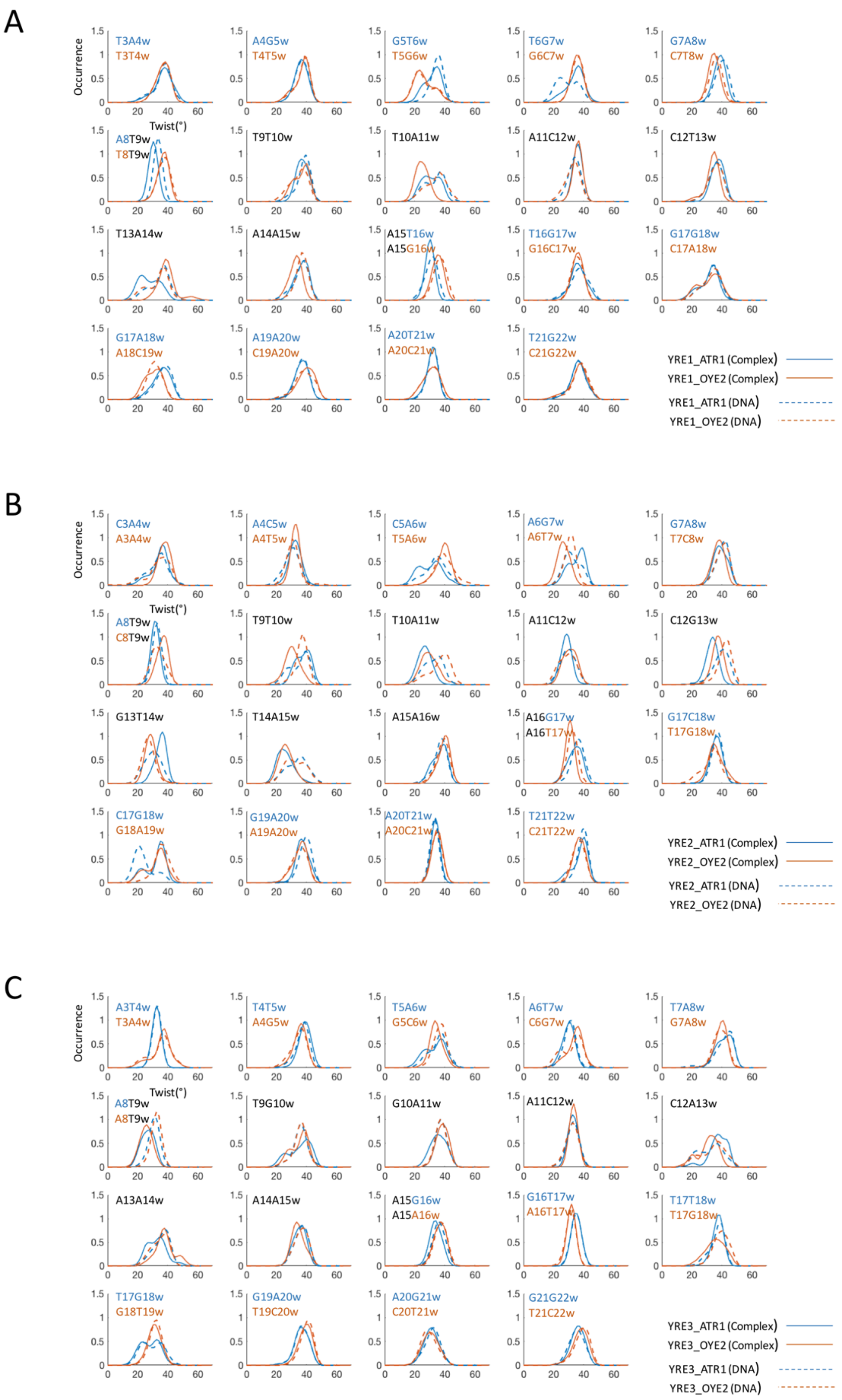

Figure S13: Normalised twist distributions for free DNA and DNA in complex with Yap1 for the three studied YREs **A.** Yap1-YRE1: TTACTAA, **B.** Yap1-YRE2: TTACGTAA, **C.** Yap1-YRE3: TGACAAA, in two genomic environments

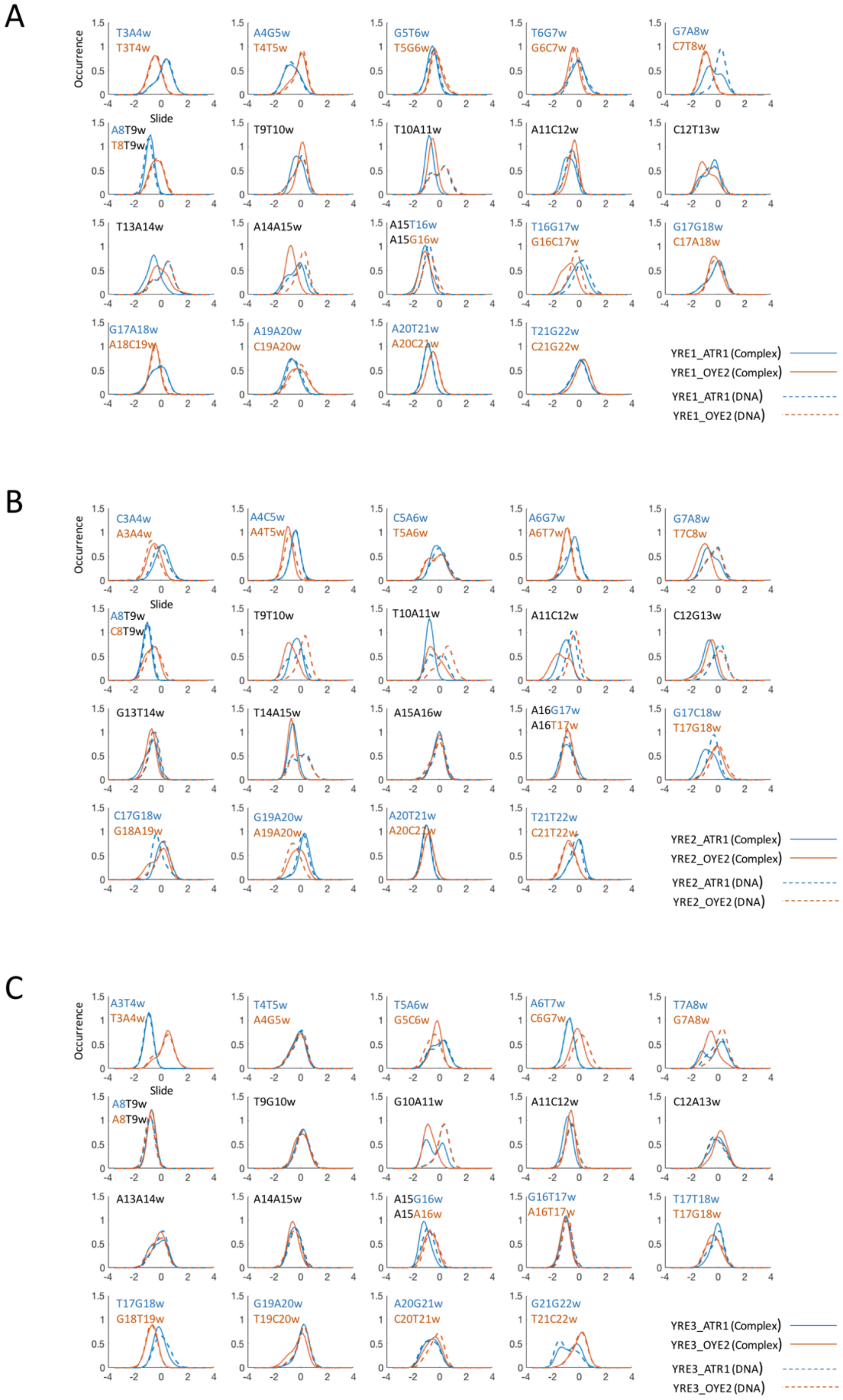

Figure S14: Normalised slide distributions for free DNA and DNA in complex with Yap1 for the three studied YREs **A.** Yap1-YRE1: TTACTAA, **B.** Yap1-YRE2: TTACGTAA, **C.** Yap1-YRE3: TGACAAA, in two genomic environments

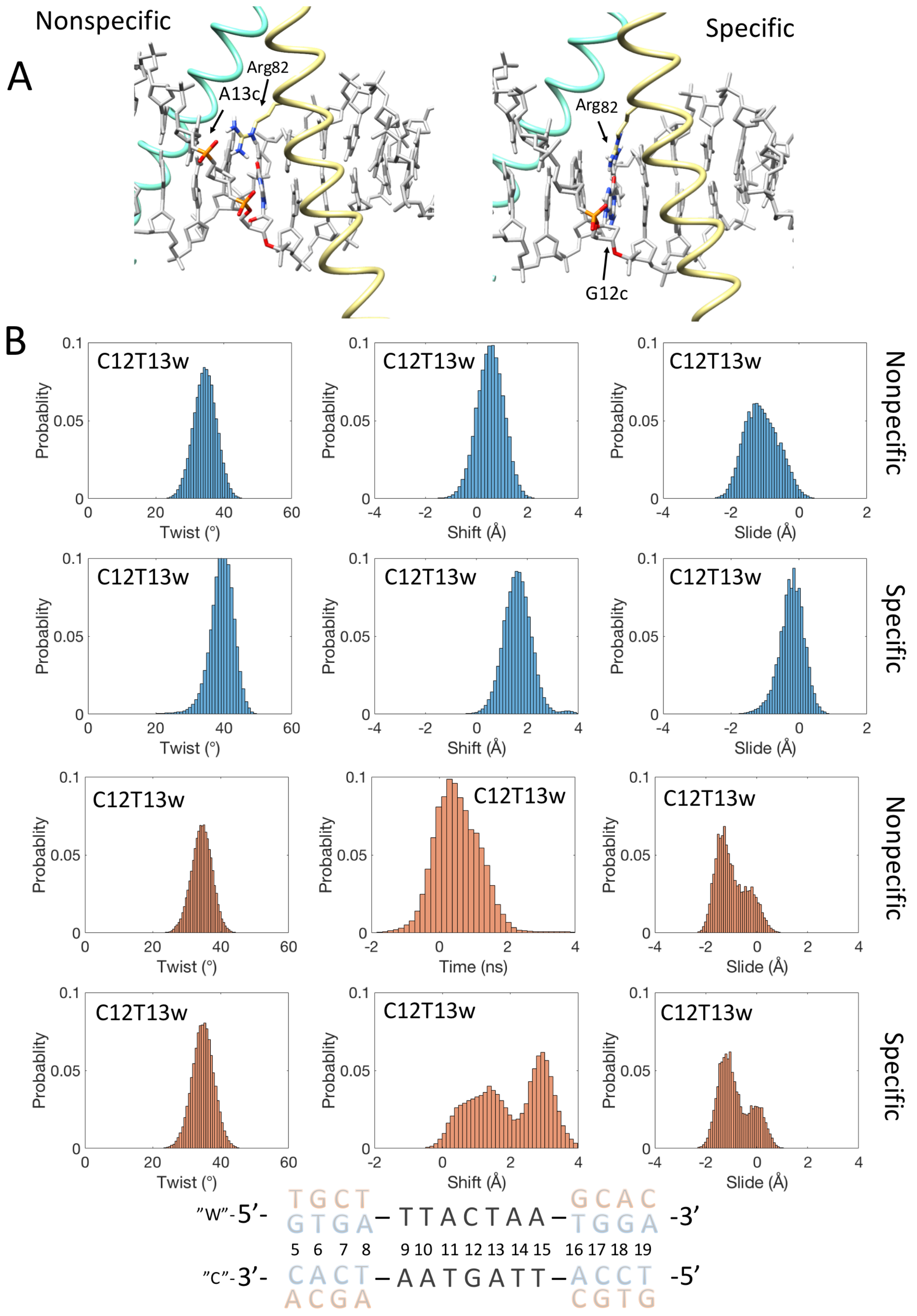

Figure S15: **A.** Nonspecific and Specific contact exploited by Yap1 Arg82 (RxxxNxxAQxxF**R**)with the central CG bp of YRE1. **B.** Twist-, Shift-, and Slide distributions for central CT bp step when Arg82 interacts non-specifically or specifically with YRE1.

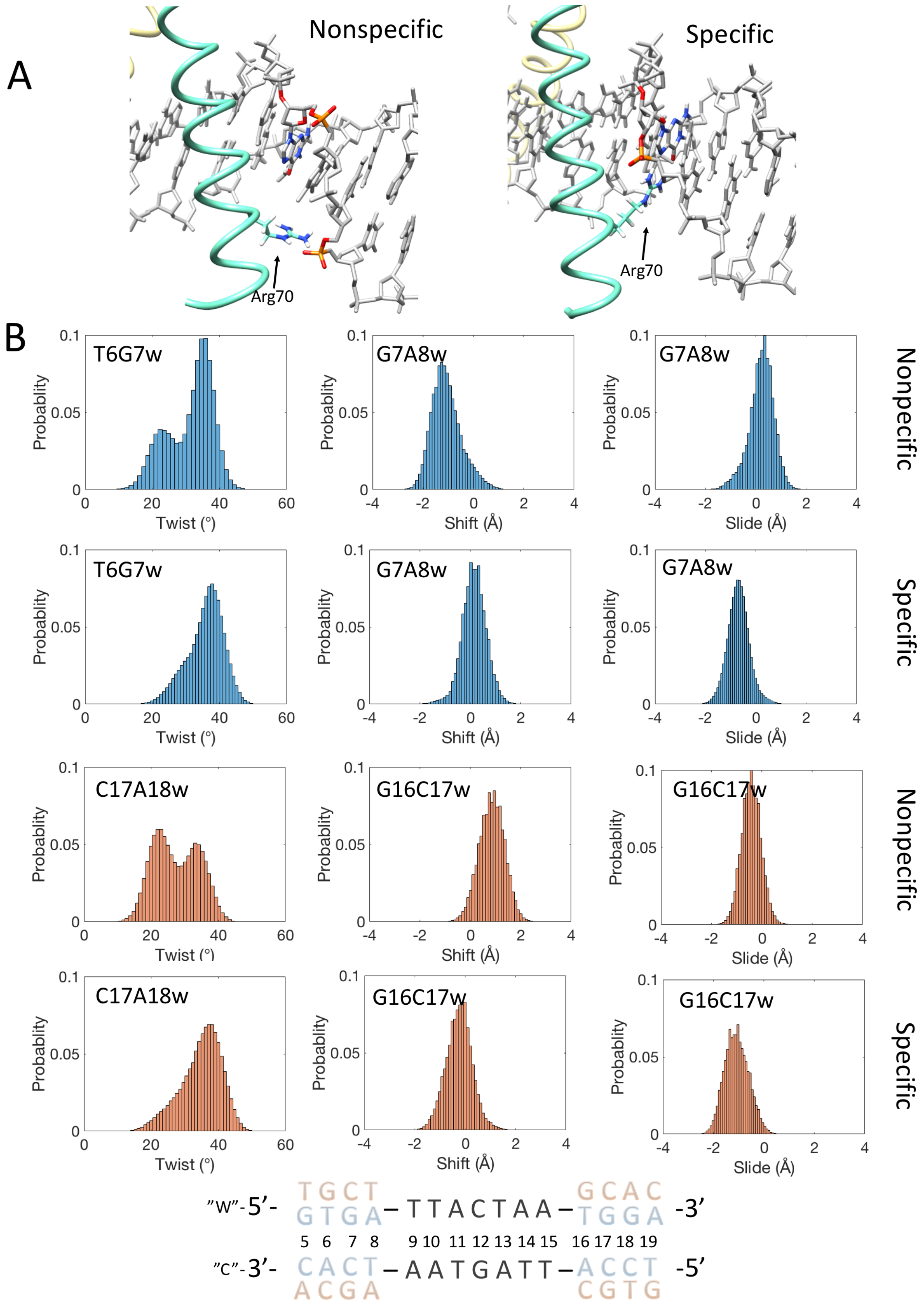
A
Nonspecific
Specific
Arg70
Arg70
B
T6G7w
G7A8w
G7A8w
Nonspecific
Specific
C17A18w
G16C17w
G16C17w
Probablity
Twist (°)
Shift (Å)
Slide (Å)
"W"-5'- TGCT GTGA – TTACTAA – GCAC TGGA -3'
5 6 7 8 9 10 11 12 13 14 15 16 17 18 19
"C"-3'- CACT ACGA – AATGATT – ACCT CGTG -5'

C

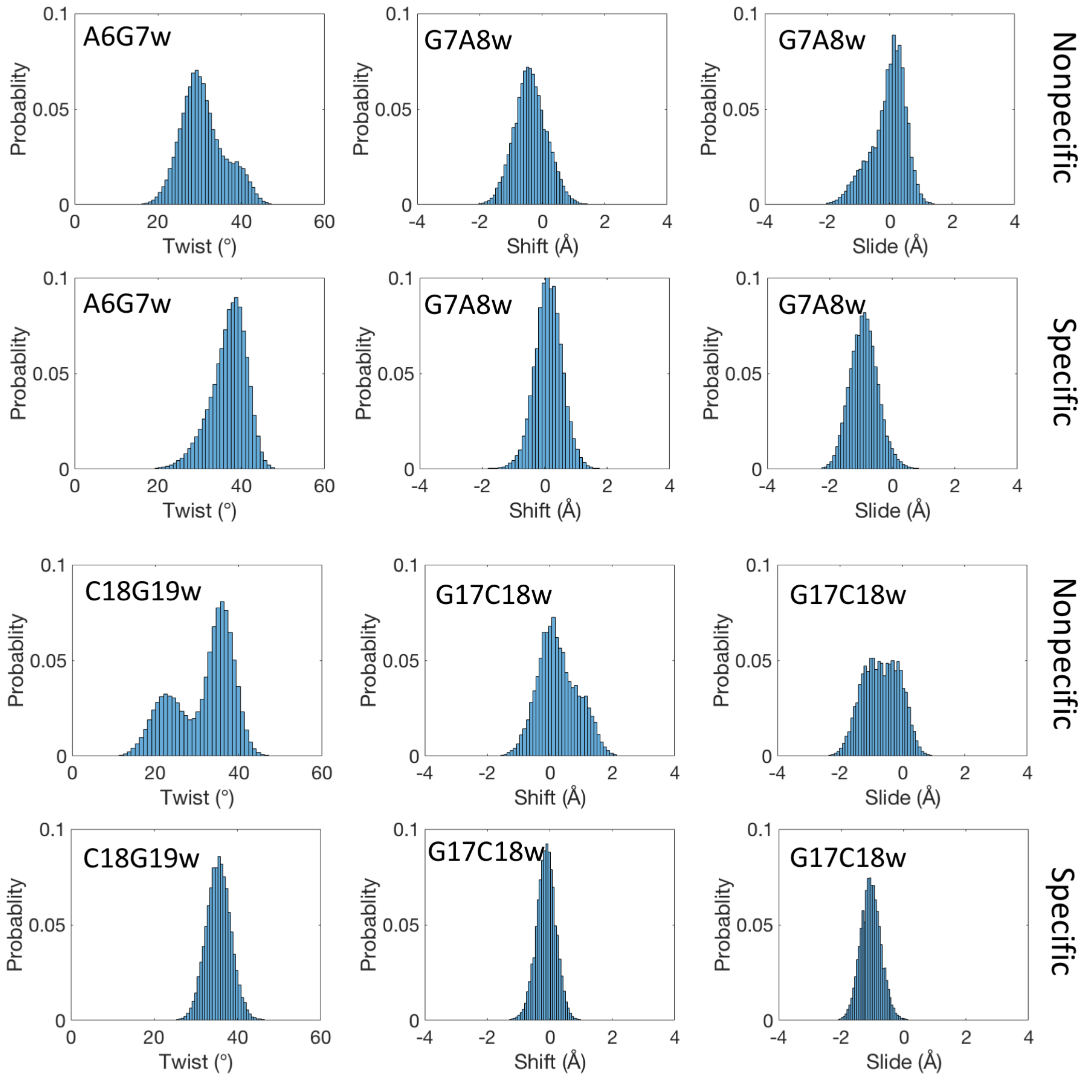

”W”-5’- C A G A – T T A C G T A A – G C G A -3’

5 6 7 8 9 10 11 12 13 14 15 16 17 18 19 20

”C”-3’- G T C T – A A T G C A T T – C G C T -5’

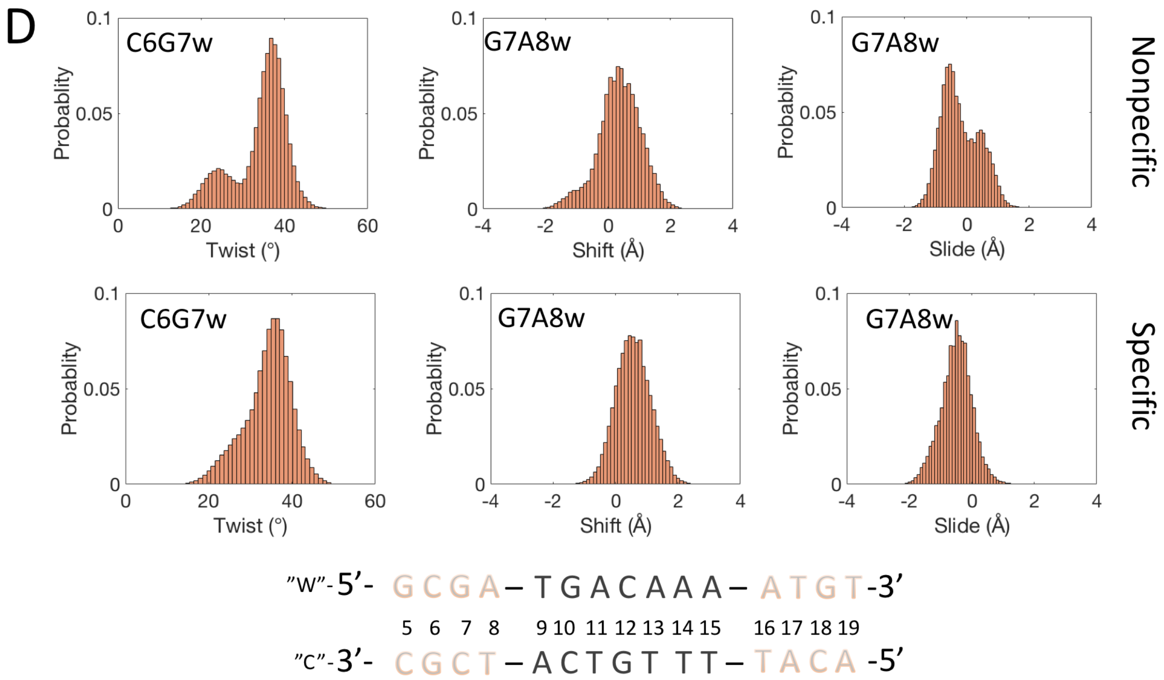

Figure S16: **A.** When Arg70 (RxxxNxxAQxxF**R**) interacts non-specifically or specifically (**G**(A/C) YRE) with DNA flanking sites. **B.** Twist-, Shift-, and Slide distributions when Arg70 interacts non-specifically or specifically with YRE1 flanking sites. **C.** Twist-, Shift-, and Slide distributions when Arg70 interacts non-specifically or specifically with YRE2-ATR1 flanking sites. **D.** Twist-, Shift-, and Slide distributions when Arg70 interacts non-specifically or specifically with YRE3-OYE2 flanking sites.

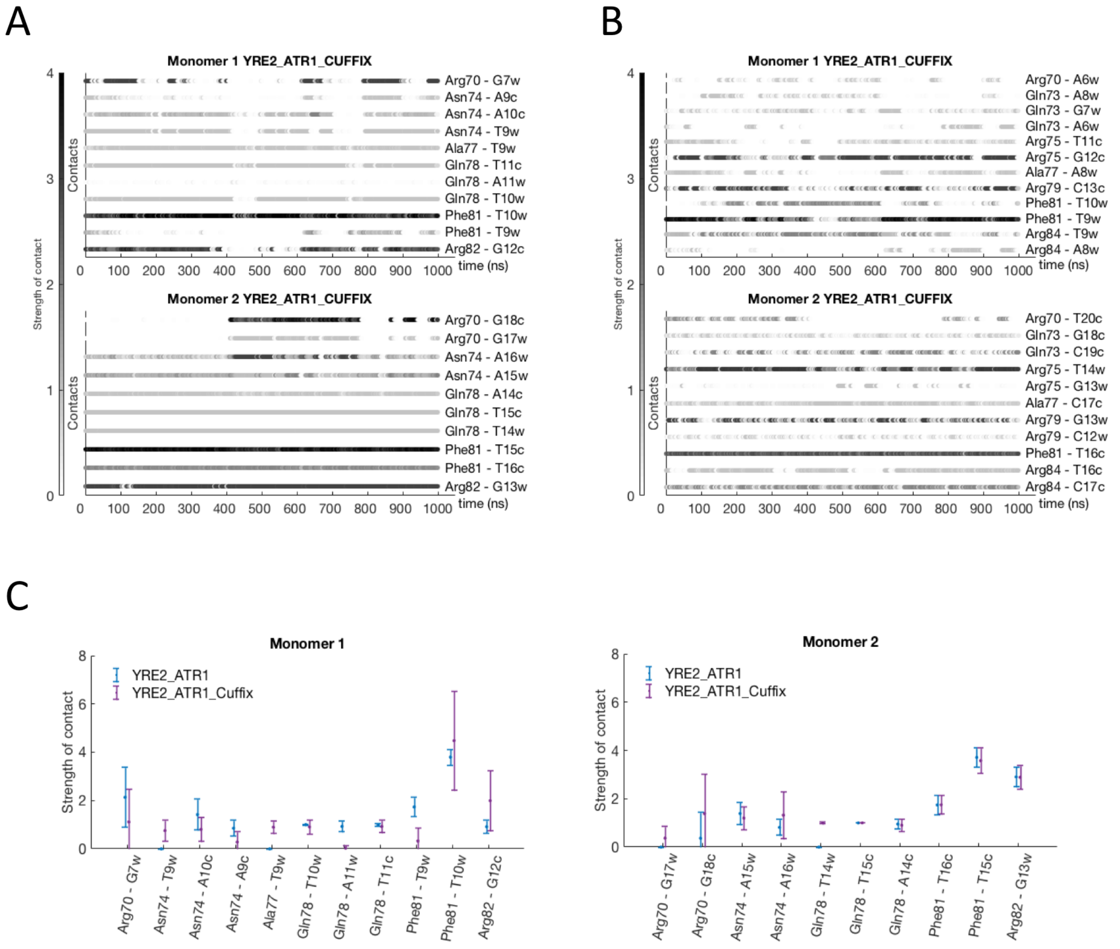

Figure S17: Yap1-YRE2_ATR1 simulation with Cufix corrections. **A.** Dynamic contact map for specific contacts and **B.** Nonspecific contacts. **C.** Differences in strength of specific contacts with (purple) and without (blue) Cuffix corrections.

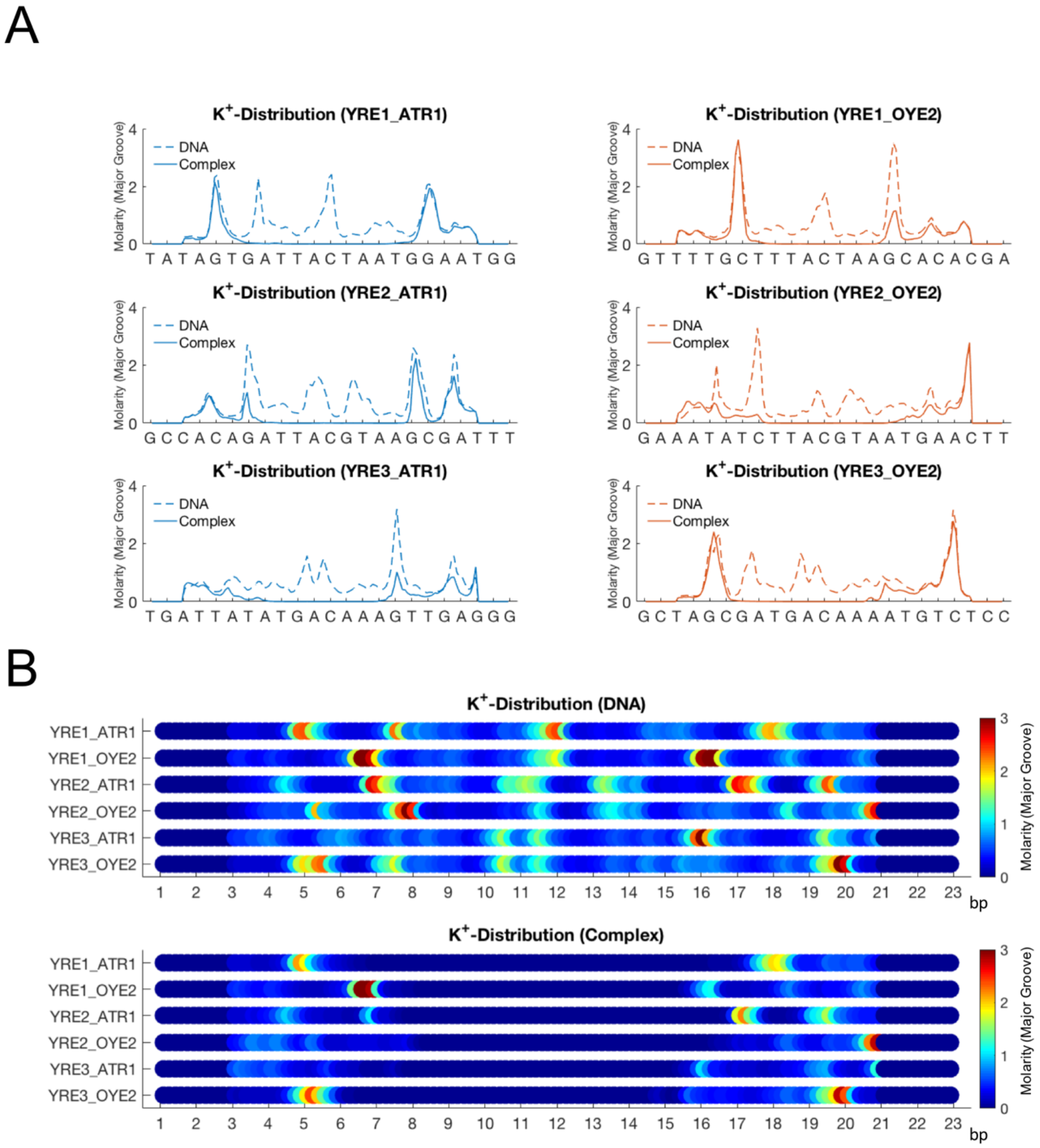

Figure S18: K+-distributions within DNA major groove along the DNA helix. **A.** Comparison of Yap1-bound DNA (thick lines) with unbound DNA (dashed lines) for the three YREs in the two genomic environments (ATR1: blue and OYE2: orange). **B.** Colormaps for the K+-distributions.

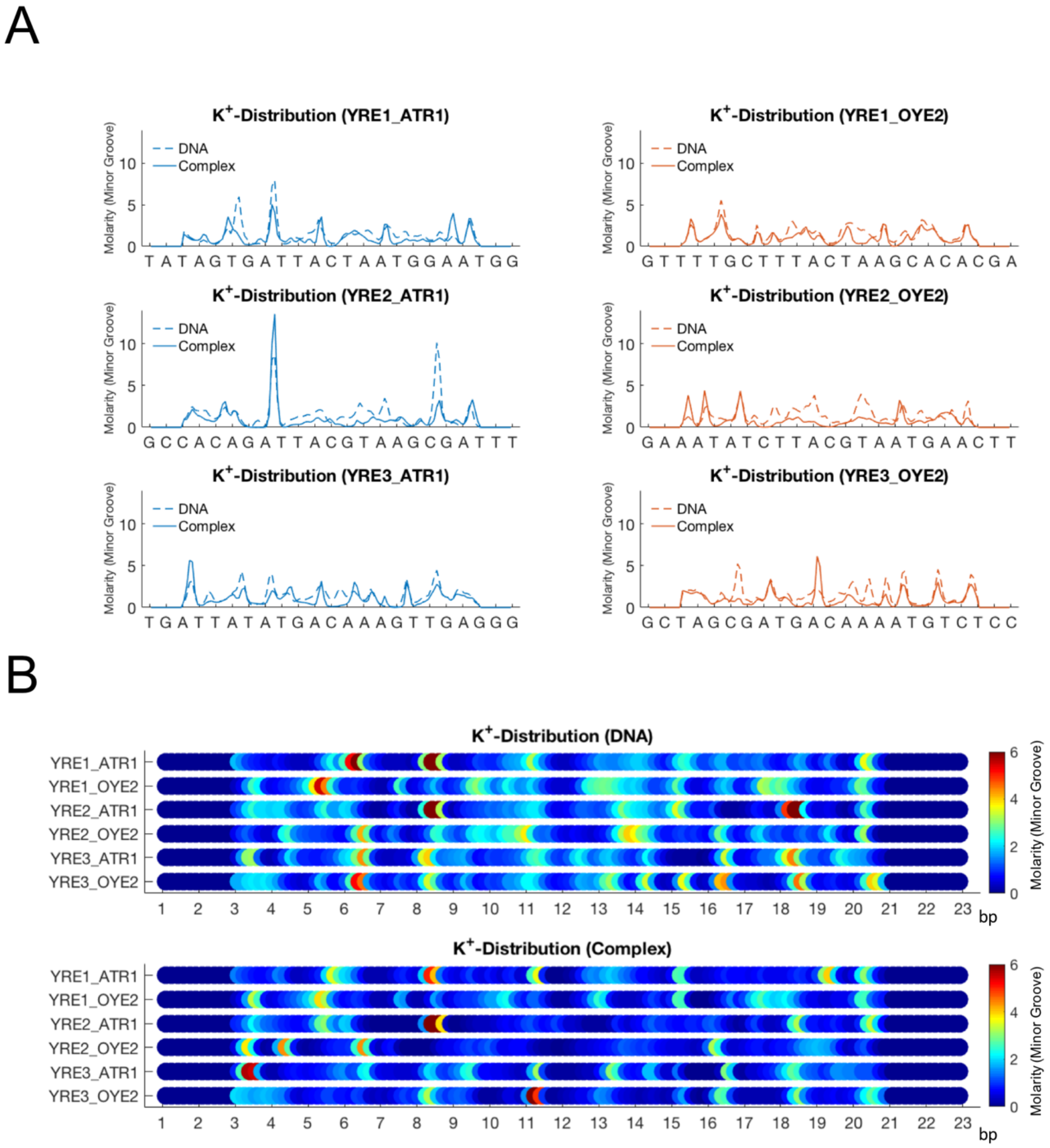

Figure S19: K+-distribution within DNA minor groove along the DNA helix. **A.** Comparison of Yap1-bound DNA (thick lines) with unbound DNA (dashed lines) for the three YREs in the two genomic environments (ATR1: blue and OYE2: orange). **B.** Colormaps for the K+-distributions.

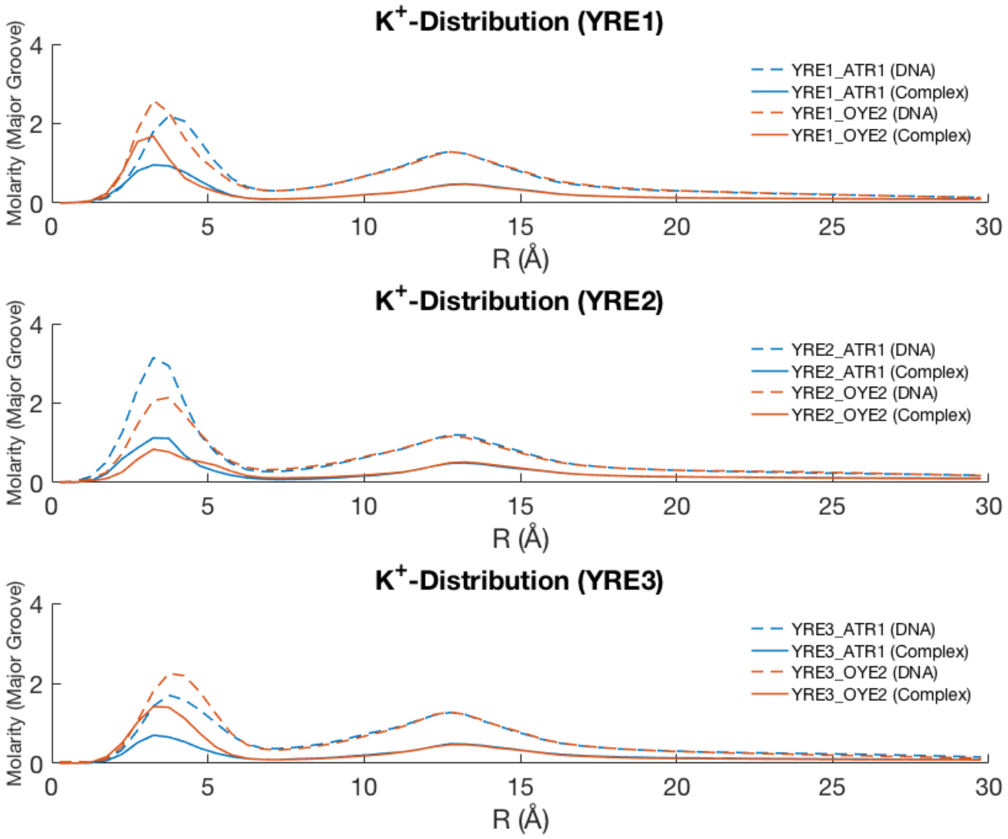

Figure S20: K+-distribution for DNA major groove at distance R (Å) from the helical axis for the three YREs (Yap1-bound DNA: thick lines, and unbound DNA: dashed lines) in the two genomic environments (ATR1: blue and OYE2: orange). A distance R<10.25 Å (phosphorous radius) constitutes the K+-molarity for the internal region, that is within the groove.

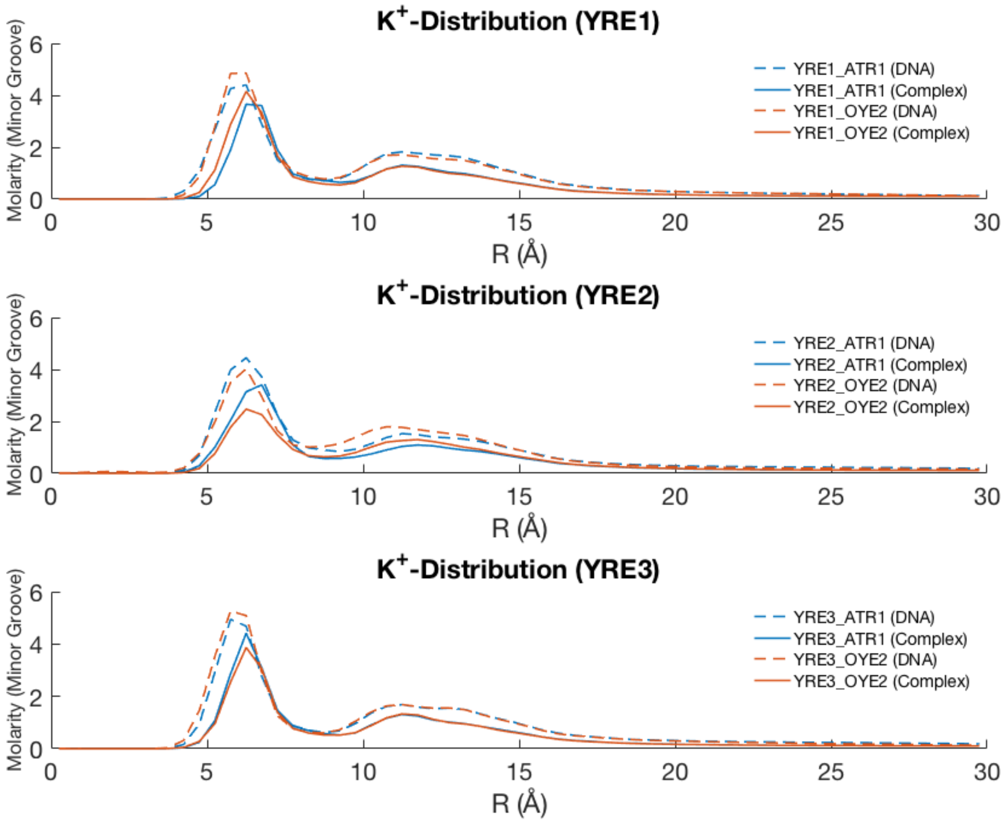

Figure S21: K+-distribution for DNA minor groove at distance R (Å) from the helical axis for the three YREs (Yap1-bound DNA: thick lines, and unbound DNA: dashed lines) in the two genomic environments (ATR1: blue and OYE2: orange). A distance R<10.25 Å (phosphorous radius) constitutes the K+-molarity for the internal region, that is within the groove.

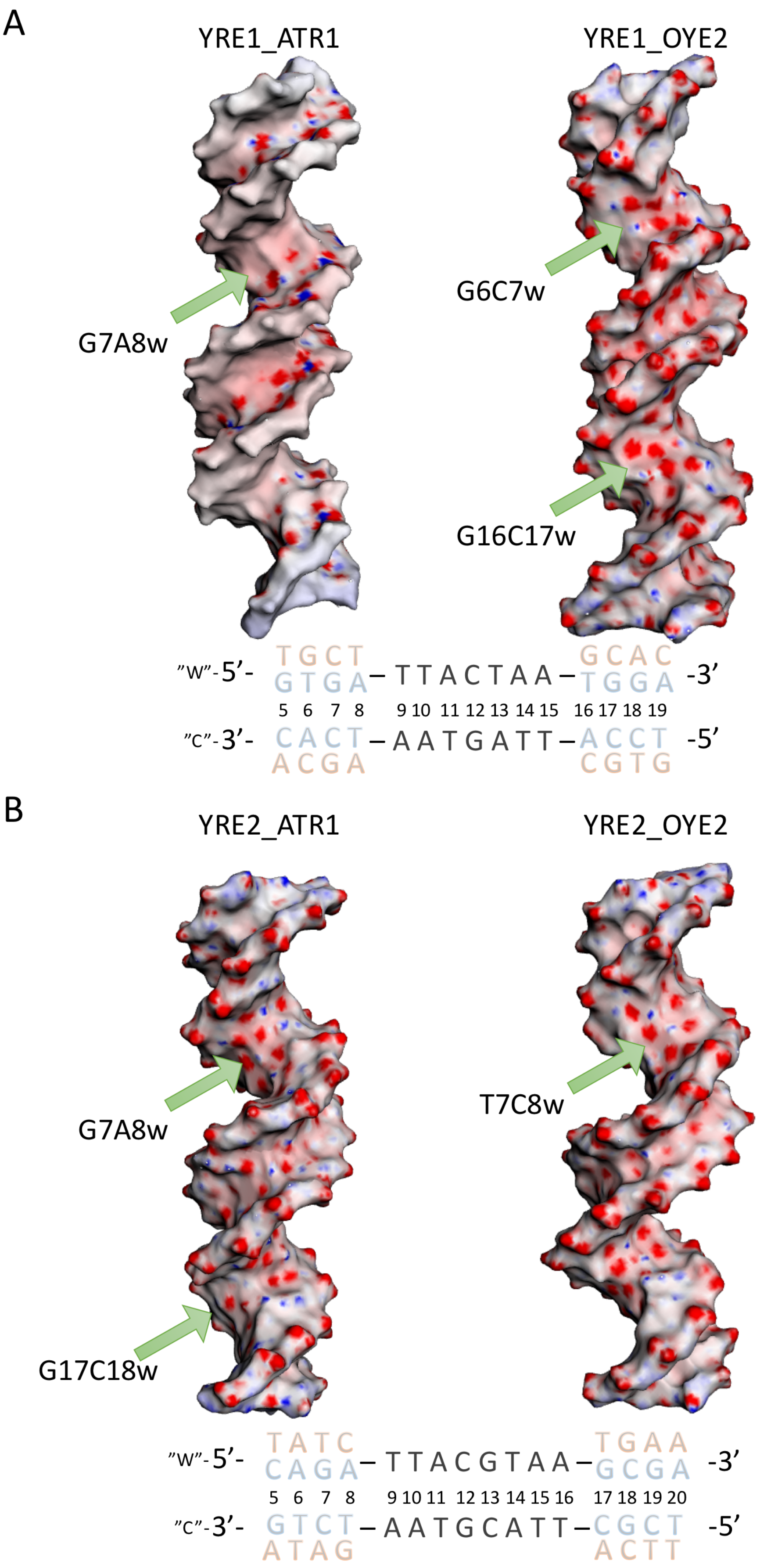
A
YRE1_ATR1
YRE1_OYE2
G7A8w
G6C7w
G16C17w
"W"-5'- TGCT GTGA – TTACTAA – GCAC TGGA -3'
5 6 7 8 9 10 11 12 13 14 15 16 17 18 19
"C"-3'- CACT ACGA – AATGATT – ACCT CGTG -5'
B
YRE2_ATR1
YRE2_OYE2
G7A8w
T7C8w
G17C18w
"W"-5'- TATC CAGA – TTACGTAA – TGAA GCGA -3'
5 6 7 8 9 10 11 12 13 14 15 16 17 18 19 20
"C"-3'- GTCT ATAG – AATGCATT – CGCT ACTT -5'

C

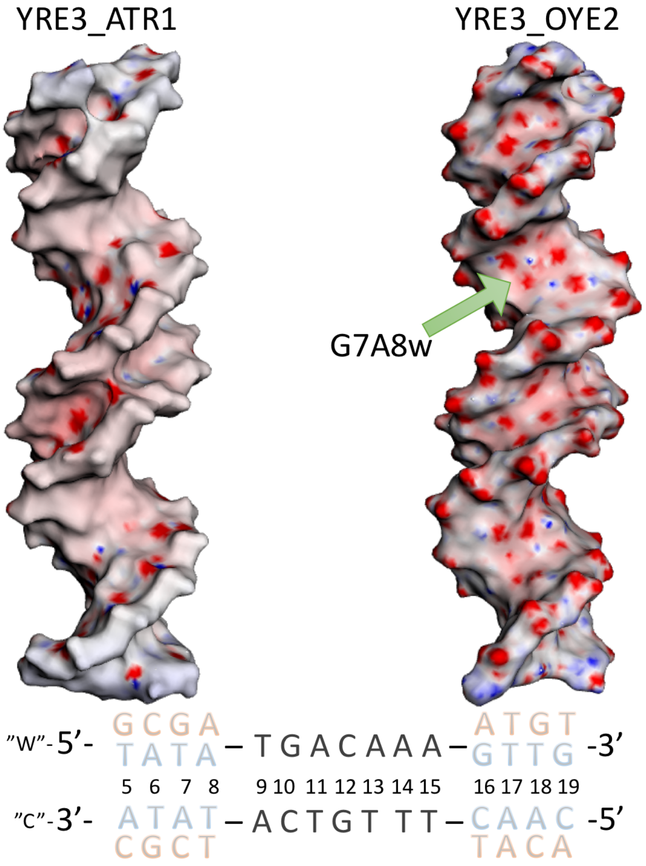

Figure S22. Derived electrostatic surfaces using APBS-PDB2QPR: A. YRE1, B. YRE2, C. YRE3. Base pair steps that show high K+-molarity by Canion are highlighted with green arrows.

Table S3: MMPBSA of Yap1-YRE complexes.

System	MMPBSA (ΔH in kcal/mol)	
	ATR1	OYE2
**YRE1**	-205±14.7	-196±12.7
**YRE2**	-213±15.6	-212±15.8
**YRE3**	-209±13.2	-213±13.7
	MMGBSA (ΔH in kcal/mol)	
	ATR1	OYE2
**YRE1**	-133±14.9	-123±14.9
**YRE2**	-147±16.9	-144±18.3
**YRE3**	-138±15.1	-142±15.2
	Entropy (ΔTS in kcal*K/mol)[a]	
	ATR1	OYE2
**YRE1**	-53.3±14.0	-65.6±14.9
**YRE2**	-65.9±10.4	-76.3±15.0
**YRE3**	-66.9±10.8	-69.5±15.6
	Free energy of binding (ΔG in kcal/mol)[b]	
	ATR1	OYE2
**YRE1**	-79.7±20.4	-57.4±21.0
**YRE2**	-81.1±19.8	-67.7±23.7
**YRE3**	-71.1±18.6	-72.5±21.8

a. T=298.15 K. b. Free energy was calculated using the equation "ΔG = ΔH – TΔS", and the derived average values for ΔH (MMGBSA) and TΔS

.

Table S4: Helical Shift values for bp step within the response element (TGACTCA, TGACGTCA, TTACGTAA) of different solved XRAY BZIP-DNA complexes with resolution "Res".

PDB	Res.(Å)	NN	NN	**TG**	**GA**	**AC**	**CT**	**TC**	**CA**	NN	NN
1FOS	3.05	TA	AT							AT	TC
		0.23<br>-0.13	-0.27<br>0.05	-0.58<br>0.06	0.37<br>-0.13	-1.12<br>0.27	0.97<br>-0.28	-0.11<br>0.12	0.54<br>-0.03	0.11<br>-0.26	-0.07<br>0.44
2H7H	2.30	GA	AT							AT	TC
		-0.06<br>-0.77	-0.01<br>-0.03	-0.63<br>0.06	0.21<br>-0.15	-0.39<br>0.28	0.66<br>-0.28	-0.36<br>0.12	0.58<br>-0.08	0.17<br>-0.06	-0.07<br>0.98
5VPF	2.69	GG	GT							AC	CC
		-0.19<br>0.18	-0.31<br>-0.41	-0.52<br>0.12	0.33<br>-0.13	-0.75<br>0.29	1.11<br>-0.29	-0.40<br>0.10	0.46<br>-0.16	0.04<br>0.40	0.34<br>-0.18
2WT7	2.30	GC	CT							AT	TA
		0.44<br>-0.17	0.03<br>-0.20	-0.43<br>0.03	0.28<br>-0.14	-0.59<br>0.25	0.92<br>-0.28	-0.68<br>0.11	0.47<br>-0.09	0.62<br>0.26	-0.42<br>-0.14
2WTY	2.90	GC	CT							AG	GC
		0.52<br>-0.16	0.00<br>-0.20	-0.87<br>0.03	0.25<br>-0.14	-0.38<br>0.26	0.33<br>-0.27	-0.13<br>0.12	0.62<br>-0.05	-0.02<br>0.17	-0.04<br>0.19
				–<br>+	+<br>–	–<br>+	+<br>–	–<br>+	+<br>–		

PDB	Res.(Å)	NN	NN	**TG**	**GA**	**AC**	**CG**	**GT**	**TC**	**CA**	NN	NN
1JNM	2.20	GA	AT								AT	TC
		0.06<br>-0.78	-0.11<br>-0.05	-0.62<br>0.08	0.14<br>-0.53	0.08<br>0.71	0.25<br>0.00	-0.17<br>-0.71	0.10<br>0.53	0.72<br>-0.08	-0.01<br>0.06	-0.13<br>0.77
1DH3	3.00	GC	CT								AG	GC
		0.39<br>0.02	0.04<br>-0.19	-0.69<br>0.06	0.05<br>-0.80	0.28<br>0.79	0.29<br>0.00	-0.31<br>-0.78	0.28<br>0.80	0.52<br>-0.06	-0.59<br>0.19	-0.10<br>-0.02
4AUW	2.90	GC	CT								AG	GC
		0.41<br>-0.16	0.09<br>-0.22	-0.46<br>0.09	0.13<br>-0.80	0.38<br>0.79	0.23<br>0.00	-0.37<br>-0.79	0.10<br>0.81	0.51<br>-0.09	-0.05<br>0.22	-0.35<br>0.20
				–<br>+	+<br>–	+<br>+	+<br>**0**	–<br>–	+<br>+	+<br>–		
	Res.(Å)	NN	NN	**TT**	**TA**	**AC**	**CG**	**GT**	**TA**	**AA**	NN	NN
1GD2	2.00	GG	GT								AC	CC
		-1.33<br>-0.07	0.16<br>-0.65	0.13<br>0.03	0.71<br>-0.02	0.40<br>0.33	0.10<br>0.00	-0.59<br>-0.33	-0.64<br>0.02	-0.23<br>-0.03	-0.01<br>0.65	0.61<br>0.07
				+<br>+	+<br>–	+<br>+	+<br>**0**	–<br>–	–<br>+	–<br>–		

Table S5: Helical shift for naked DNA for each bp step in each tetranucleotide environment of the response elements, TGACTCA, TGACGTA, TTACGTAA. The parameters have been collected from the BIGNASim database (http://mmb.irbbarcelona.org/BIGNASim/), which have been derived through microsecond long MD simulations of DNA dodecamers.

N	**TG**	**GA**	**AC**	**CT**	**TC**	**CA**	N	
A	0.62±1.1	-1.0±0.68	0.042±0.59	0.20±0.70	0.60±0.81	-0.62±1.1	A	
C	0.28±0.96					-0.28±0.96	C	
T	0.36±1.0					-0.36±1.0	T	
G	1.0±0.88					-1.0±0.88	G	
N	**TG**	**GA**	**AC**	**CG**	**GT**	**TC**	**CA**	N
A	0.62±1.1	-1.0±0.68	0.46±0.65	0.33±0.86	-0.46±0.65	1.0±0.68	-0.62±1.1	A
T	0.28±0.96						-0.28±0.96	T
C	0.36±1.0						-0.36±1.0	C
G	1.0±0.88						-1.0±0.88	G
N	**TT**	**TA**	**AC**	**CG**	**GT**	**TA**	**AA**	N
A	0.87±0.65	-0.83±1.1	0.23±0.90	0.33±0.86	-0.23±0.90	0.83±1.1	-0.87±0.65	A
C	0.43±0.71						-0.43±0.71	C
T	0.41±0.77						-0.41±0.77	T
G	1.0±0.65						-1.0±0.65	G

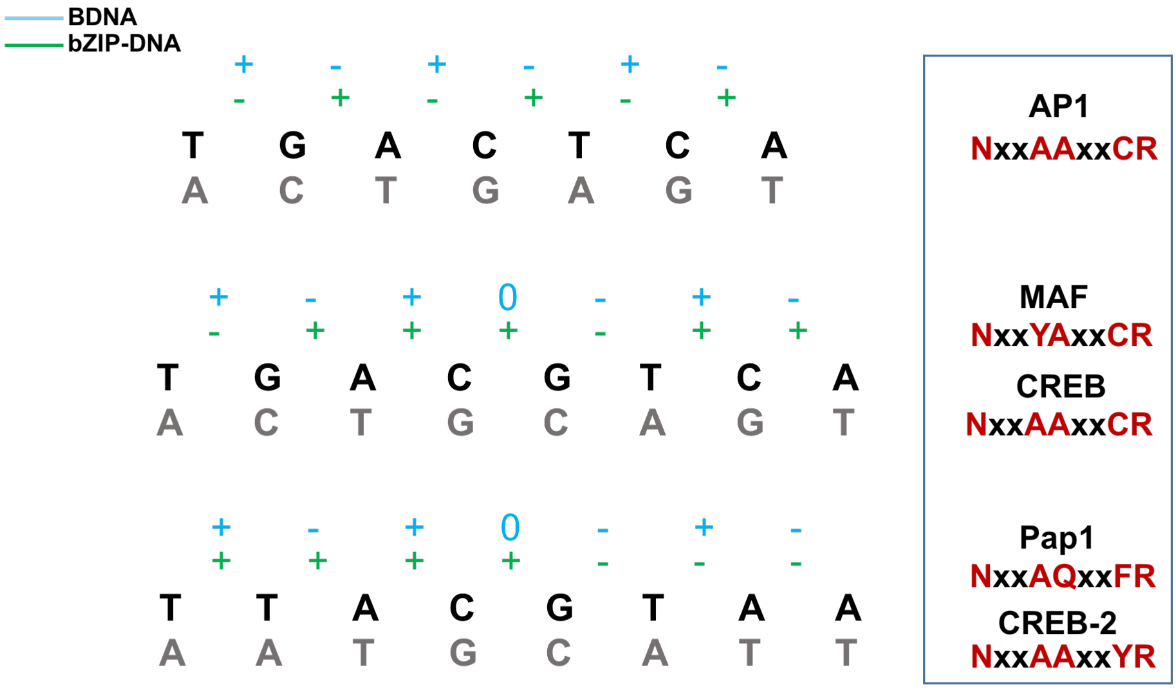

Figure S23: The sign of helical shift for b.p. steps within the three response elements for bZIP-bound DNA (derived from crystal structures) and B-DNA (derived from modelling tool JUMNA). The box to the right shows BZIP families (with the five-residue motif highlighted) that recognise the different response elements.

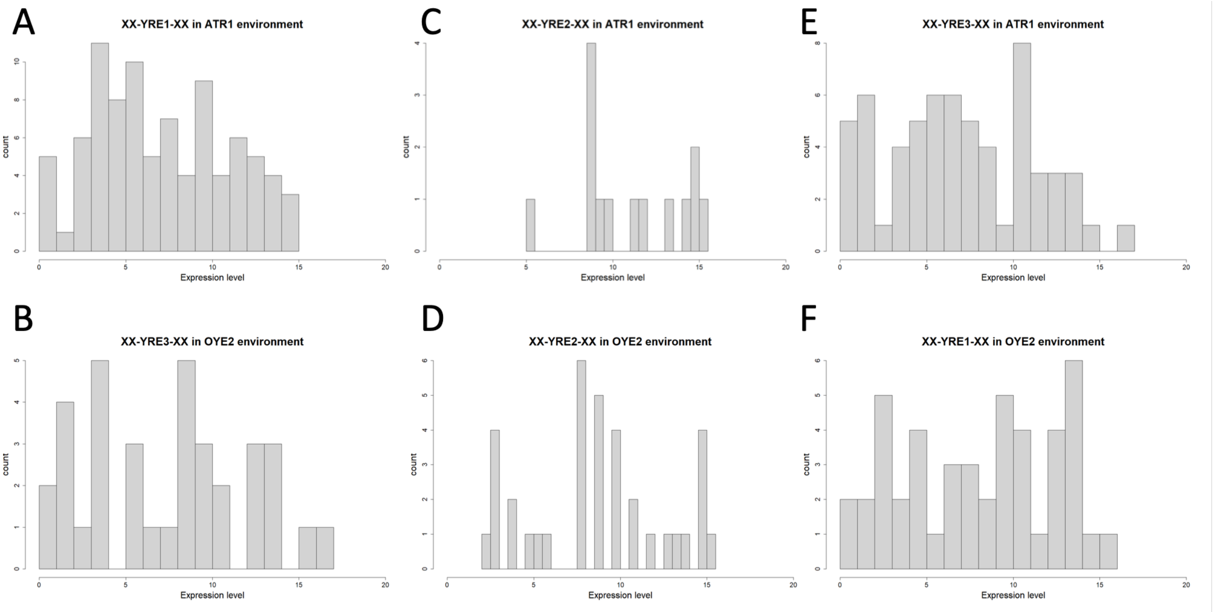

Figure S24: Expression levels distributions for all unique groups NN-YRE-NN, containing either the two adjacent flanking nucleotides (denoted as "N") as is in the ATR1- or OYE2-environments. **A.** YRE1_ATR1 **B.** YRE1_OYE2 **C.** YRE2_ATR1 **D.** YRE2_OYE2 **E.** YRE3_ATR1 **F.** YRE3_OYE2.

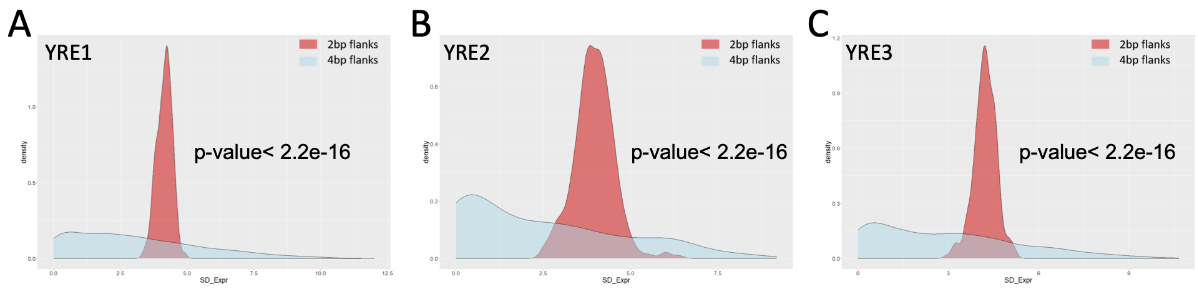

Figure S25: Comparison of the expression levels standard deviations distributions for unique groups of NN-YRE[1/2/3]-NN versus NNNN-YRE[1/2/3]-NNNN filtered promoters. Filtered promoter sequences from 80-b.p. model promoter dataset,[18] containing only one known TF-binding site according to YeTFaSCo database[19], have been divided into unique groups based on the combination of two adjacent flanking nucleotides, yielding 256 groups for each YRE, with the following total number of promoter sequences: for YRE1 – 17371, YRE2 – 4952, and YRE3 – 11020; and for four adjacent flanking nucleotides, yielding for YRE1 – 14315 groups containing 17371 sequences; YRE2 – 4595 groups and 4952 sequences, and YRE3 – 9644 groups and 11020 sequences, correspondingly. Thereafter, for each unique group with more than one sequence, the standard deviations of the expression levels are calculated, followed by the Student t-test analysis showing a significant difference in the distributions of expression level standard deviations between the two selected sets of promoters.

```
Yeast_Yap1           -KQKRTAQNRAAQRAFRERKERKMKELEKKVQSLESIQQQNEVEATFLRDQLITL-----
Mouse_CREB/ATF       TKSPRK-AAAAAARLNRLKKKEYVMGLESRVRGLAAE-------NQELRAENRELGKRVQ
C_elegans_ZIP-4      YKLKRA-RNNDAVRKSRNKAKELQLQKDEEYDEMKKR-------ITQLEAELQSEREGRE
Arabidopsis          RHKRMI-KNRESAARSRARKQAYTNELELEVAHLQAE-------NARLKRQQDQLKMAAA
Human_C_Fos          RRIRRE-RNKMAAAKSRNRRRELTDTLQAETDQLEDE-------KSALQTEIANLLKEKE
Chicken_E4BP4        YWEKRR-KNNEAAKRSREKRRLNDLVLENKLIALGEE-------NATLKAELLSLKLKFG
Norway_rat_HLF       YWARRR-KNNMAAKRSRDARRLKENQIAIRASFLEKE-------NSALRQEVADLRKELG
```

Figure S26: Multiple sequence alignment of BZIP proteins.
